# CGRP and the Calcitonin Receptor are Co-Expressed in Mouse, Rat and Human Trigeminal Ganglia Neurons

**DOI:** 10.3389/fphys.2022.860037

**Published:** 2022-05-10

**Authors:** Tayla A. Rees, Andrew F. Russo, Simon J. O’Carroll, Debbie L. Hay, Christopher S. Walker

**Affiliations:** ^1^ School of Biological Sciences, University of Auckland, Auckland, New Zealand; ^2^ Maurice Wilkins Centre for Molecular Biodiscovery, University of Auckland, Auckland, New Zealand; ^3^ Department of Molecular Physiology and Biophysics, Center for the Prevention and Treatment of Visual Loss, Veterans Administration Health Center, Department of Neurology, University of Iowa, Iowa City, IA, United States; ^4^ Department of Anatomy and Medical Imaging and Centre for Brain Research, Faculty of Medical and Health Science, University of Auckland, Auckland, New Zealand; ^5^ Department of Pharmacology and Toxicology, University of Otago, Dunedin, New Zealand

**Keywords:** CGRP—calcitonin gene-related peptide, migraine, headache, G protein-coupled receptor, trigeminal ganglia, amylin, amylin 1 (AMY_1_)

## Abstract

The neuropeptide calcitonin gene-related peptide (CGRP) is expressed in the trigeminal ganglia, a key site in craniofacial pain and migraine. CGRP potently activates two receptors: the CGRP receptor and the AMY_1_ receptor. These receptors are heterodimers consisting of receptor activity-modifying protein 1 (RAMP1) with either the calcitonin receptor-like receptor (CLR) to form the CGRP receptor or the calcitonin receptor (CTR) to form the AMY_1_ receptor. The expression of the CGRP receptor in trigeminal ganglia has been described in several studies; however, there is comparatively limited data available describing AMY_1_ receptor expression and in which cellular subtypes it is found. This research aimed to determine the relative distributions of the AMY_1_ receptor subunit, CTR, and CGRP in neurons or glia in rat, mouse and human trigeminal ganglia. Antibodies against CTR, CGRP and neuronal/glial cell markers were applied to trigeminal ganglia sections to investigate their distribution. CTR-like and CGRP-like immunoreactivity were observed in both discrete and overlapping populations of neurons. In rats and mice, 30–40% of trigeminal ganglia neurons displayed CTR-like immunoreactivity in their cell bodies, with approximately 78–80% of these also containing CGRP-like immunoreactivity. Although human cases were more variable, a similar overall pattern of CTR-like immunoreactivity to rodents was observed in the human trigeminal ganglia. CTR and CGRP appeared to be primarily colocalized in small to medium sized neurons, suggesting that colocalization of CTR and CGRP may occur in C-fiber neurons. CGRP-like or CTR-like immunoreactivity were not typically observed in glial cells. Western blotting confirmed that CTR was expressed in the trigeminal ganglia of all three species. These results confirm that CTR is expressed in trigeminal ganglia neurons. The identification of populations of neurons that express both CGRP and CTR suggests that CGRP could act in an autocrine manner through a CTR-based receptor, such as the AMY_1_ receptor. Overall, this suggests that a trigeminal ganglia CTR-based receptor may be activated during migraine and could therefore represent a potential target to develop treatments for craniofacial pain and migraine.

## 1 Introduction

Migraine is one of the most disabling neurological conditions and is estimated to affect 15–20% of people worldwide (Agosti, 2018). The discovery of calcitonin gene-related peptide (CGRP), a neuropeptide with potent vasodilatory and neuromodulatory activity, and its role in migraine pathogenesis led to the development of several breakthrough therapeutics (Edvinsson et al., 2018). These include humanized antibodies and small molecules which target CGRP and its canonical receptor, the CGRP receptor, to reduce receptor activation and signaling in migraine relevant structures (Dubowchik et al., 2020).

The precise pathophysiology of migraine is unclear, however, the trigeminovascular system appears to play a major role in the generation, processing, and modulation of migraine pain, particularly the trigeminal ganglia (TG) and the trigeminal nerves (TN) (May and Goadsby, 1999; Buzzi et al., 2003; Edvinsson J. C. A. et al., 2020). The trigeminovascular system is part of the peripheral nervous system, located outside the blood-brain barrier, and can be modulated by circulating or locally released molecules. CGRP and the molecular subunits of the canonical CGRP receptor, the calcitonin receptor-like receptor (CLR) and receptor activity-modifying protein 1 (RAMP1), are all expressed within the TG where they may contribute to migraine pathogenesis (Eftekhari et al., 2010; Eftekhari et al., 2013; Eftekhari et al., 2015; Hay et al., 2018; Rees et al., 2021b).

The AMY_1_ receptor, which comprises RAMP1 together with the calcitonin receptor (CTR), is another CGRP-responsive receptor that can be potently activated by CGRP and amylin (Bower et al., 2018; Hay et al., 2018). AMY_2_ and AMY_3_ receptors use RAMP2 or RAMP3 with CTR to form other amylin receptor subtypes (Bower et al., 2018; Hay et al., 2018). Amylin, a peptide hormone secreted from pancreatic islet β-cells in response to food intake, is closely related to CGRP (Hay et al., 2015). A recent provocation study demonstrated that infusion of an amylin analogue, pramlintide, was sufficient to induce headache and migraine-like attacks in migraineurs (Ghanizada et al., 2021). This highlights a possible role for amylin-responsive receptors, like the AMY_1_ receptor, in headache and migraine pathogenesis, through peripheral migraine-relevant structures such as the TG.

Pharmacological and protein data suggest that CTR and RAMP1 are expressed in the TG. Both subunits are co-expressed in some neurons, indicating potential expression of the AMY_1_ receptor (Walker et al., 2015; Bohn et al., 2017; Edvinsson L. et al., 2020). However, mRNA studies of CTR expression are conflicting, with little mRNA detected (Manteniotis et al., 2013; Flegel et al., 2015; Walker et al., 2015; LaPaglia et al., 2018; Edvinsson L. et al., 2020). This lack of correlation between protein and mRNA may be because mRNA abundance is not necessarily proportionally linked to protein expression (Vogel et al., 2010; Vogel and Marcotte, 2012; Gingell et al., 2020). Transcriptome and proteomic studies propose that translation efficiency and the rate of protein degradation make a significant contribution to overall protein expression levels (Vogel et al., 2010; Vogel and Marcotte, 2012). Additionally, G protein-coupled receptors (GPCR) like the CTR have highly amplified and carefully regulated signal transduction cascades and do not need to be expressed at high levels to produce functional effects. Measures of protein expression with validated antibodies are therefore important. Previous immunohistochemical studies localizing CTR in the TG used antibodies that were uncharacterized or later found to be unable to detect CTR in a particular species (Walker et al., 2015; Edvinsson L. et al., 2020; Hendrikse et al., 2022). Therefore, it is necessary to conduct additional studies with well-validated, species-appropriate antibodies to substantiate these prior reports.

Several studies report that CGRP can promote its own expression in an autocrine manner. In the TG, this autoregulatory mechanism has been linked to migraine chronification (Zhang et al., 2007; Guo et al., 2020). However, the underlying mechanism by which CGRP exerts this effect is unknown because CLR and CGRP tend to be expressed in distinct neuronal subpopulations (Lennerz et al., 2008; Eftekhari et al., 2013; Edvinsson et al., 2019). This suggests that other CGRP-responsive receptors, such as the AMY_1_ receptor, could instead act as an autoregulatory CGRP-receptor. Of note, there are species differences in the number of potential CGRP-responsive receptors. More rat and mouse CLR or CTR-based receptors are responsive to CGRP, as compared to their human counterparts (Hay et al., 2003; Bailey et al., 2012; Bohn et al., 2017; Garelja et al., 2021a). Therefore, it is important to understand where CTR might be found in relation to CGRP-expressing structures in pre-clinical model species, and in humans.

The present study was therefore designed to investigate the protein expression of the CTR, and its spatial relationship to CGRP expression in the TG of rats, mice and humans using well-validated antibodies. Histology was complimented with immunoblotting to provide an orthogonal method for determining the presence of the CTR in the TG.

## 2 Materials and Methods

### 2.1 Antibodies

All primary and secondary antibodies are detailed in Supplementary Table S1.

### 2.2 Plasmids

The N-terminally HA-tagged human CLR and CTR (CT_(a)_ splice variant, leucine polymorphic variant), myc- or untagged human RAMP1 constructs in pcDNA3.1 were as previously described (Qi et al., 2013). Untagged rat CLR, CTR and RAMP1 constructs in pCMV6 were from Origene (Rockville, United States). The untagged mouse CLR, CTR and RAMP1 in pCMV6 plasmids were as previously described (Garelja et al., 2021a). Rat and mouse CTR were the CT_(a)_ variant.

### 2.3 Cell Culture and Transfection

HEK293S cells were cultured and transfected as previously described (Gingell et al., 2020). Cells were plated into poly-D-lysine coated Cell-Carrier Ultra plates (PerkinElmer, Waltham, MA) at 10,000 cells per well and transfected 36 h after plating with 0.25 µg of DNA, using polyethylenimine as previously described (Gingell et al., 2020). Immunocytochemistry was performed 24–36 h after transfection. For western blotting, HEK293S cells were grown in 15 cm^2^ dishes and transfected with 60 µg of DNA when ∼70% confluent. Cells were harvested for whole cell lysate preparations 48 h after transfection. In all cases, CLR or CTR were transfected in a 1:1 ratio with either RAMP1 or pcDNA3.1. Rat, mouse and human CT_(a)_ splice variants were used as controls for immunocytochemistry and immunoblotting. However, the antigenic sequences for mAb8B9, pAb188 and mAb31-01 are present in both the CT_(a)_ and CT_(b)_ variants. Therefore, these antibodies are expected to detect both variants.

### 2.4 Immunocytochemistry

Transfected HEK293S cells were fixed with 4% paraformaldehyde (PFA) and washed with Tris-buffered saline (TBS) containing 0.1% Tween20 (TBS-T). Cells were blocked with 10% donkey serum (Abcam, Cambridge, United Kingdom) in TBS-T for 1 h at room temperature (RT). Cells were then incubated with primary antibody (Supplementary Table S1) in 1% serum/TBS-T overnight at 4°C. Cells were washed twice with TBS-T then incubated with secondary antibody (1:200, Supplementary Table S1) and DAPI (ThermoFisher Scientific, Waltham, MA) in 1% serum/TBS-T for 1 h at RT. Cells were then washed twice in TBS-T and imaged.

### 2.5 Peptides

All peptides were synthesized as previously described (Bower et al., 2018; Ghanizada et al., 2021). Human αCGRP (hαCGRP) and rat αCGRP (rαCGRP) were made as 1 mM stocks in water. Human amylin (hAmy) and rat amylin (rAmy) were made up as previously described as 13 mM stocks in 100% DMSO or water, respectively (Bower et al., 2018; Ghanizada et al., 2021). All peptides were stored as aliquots in protein LoBind tubes (022431081, Eppendorf, Hamburg, DE) at−30°C.

### 2.6 Immunoblotting (Dot Blotting)

Dot blotting was performed as previously described (Rees et al., 2021a). Briefly, stock solutions of hAmy, rAmy, hαCGRP, and rαCGRP peptides were serially diluted in sterile water to give the required concentrations. The species and peptide order were randomized. Two microliters containing the total amount of each peptide required was loaded as a single spot on 0.45 µm nitrocellulose membranes (Bio-Rad, Hercules, CA). Membranes were then incubated for 1 h at RT in TBS-T with 5% (w/v) low-fat milk (assay buffer). This buffer was removed, and the membranes were then incubated with primary anti-CGRP antibodies (Supplementary Table S1) diluted in assay buffer for 1 h at RT. Membranes were then washed twice for 5 min in TBS-T and incubated with secondary antibodies diluted 1:1,000 in assay buffer for 1 h at RT. Membranes were then washed twice before the blots were developed with SuperSignal West Pico PLUS (34577, ThermoFisher Scientific) for ∼5 min. Blots were imaged using an Amersham A600 imager (GE Healthcare, Chicago, IL). Image acquisition was performed using the automated exposure function with the high dynamic range setting. All blots presented are representative of consistent results from at least three independent experiments.

As the 100 µg hAmy stock was dissolved in 100% DMSO, comparisons were made to 100% DMSO alone to determine whether it affected immunoreactivity. This was performed on both nitrocellulose and polyvinylidene fluoride (PVDF) membranes (Cat# LC 2,005 Life Technologies, Carlsbad, CA). For nitrocellulose membranes, the addition of 2 µl of 100 µg hAmy (in 100% DMSO) or 100% DMSO alone was performed as above. For the PVDF membranes, 2 µl of hAmy (in 100% DMSO) or 100% DMSO alone was added to membranes pre-wet with methanol. Fifty ng of rαCGRP was included as a positive control. Membranes were then incubated at RT for approximately 5 min while being kept moist with TBS-T. Membranes were then incubated with assay buffer and immunoblotting was performed as above.

### 2.7 Tissue Collection—Mouse and Rat

All procedures involving the use of animals were conducted in accordance with the New Zealand Animal Welfare Act (1999) and approved by the University of Auckland Animal Ethics Committee. Rodents of the same sex were housed with littermates in Tecniplast Greenline IVC with Sealsafe Plus GM500 cages (mice) or as pairs in Teciplast Conventional 1500U cages (rats) in a controlled environment (12-h light-dark cycle; room temperature, 22 ± 2°C) with ad libitum access to standard chow (Teklad TB 2018; Harlan, Madison, WI) and water. Cages also contained an additional enrichment item (house or toy). Tissue was collected from available animals culled as part of routine colony maintenance. The estrous cycle phase was not assessed or recorded for female rats or mice. Animal details are provided in Supplementary Table S2.

Anesthesia was induced with 5% isoflurane in 2 L/min O_2_, and the animals euthanized by cervical dislocation. Tissues were dissected quickly from male and female Sprague-Dawley (SD) rats and C57BL/6J mice. Tissues collected for western blotting were snap-frozen in liquid nitrogen and stored at −80°C. Tissues collected for immunohistochemistry were washed with phosphate-buffered saline (PBS) and placed in 4% PFA for 24 h at 4^°^C. After fixation, tissues were cryoprotected with 10%, then 20% sucrose (w/v) in PBS and embedded in optimal cutting temperature compound (Sakura Tissue-Tek, 4583). Pancreata and TG were cryo-sectioned transversely or sagittally, respectively, at a thickness of 12 µm using a Leica CM1850 microtome (Leica Biosystems, Wetzlar, Germany). Sections were mounted onto slides and then stored at −80°C.

### 2.8 Tissue Collection—Human

For immunohistochemistry, postmortem human TG were obtained from the University of Auckland Human Anatomy Laboratory, with informed consent by the donor before death and next of kin after death as part of the University of Auckland Human Body Bequest Program for teaching and research. This program and its procedures operate under the Human Tissue Act of 2008 and are overseen by the New Zealand Police Inspector of Anatomy. For western blotting, fresh-frozen postmortem human TG and trigeminal nerve (TN) was obtained from the NIH NeuroBioBank. Case details are provided in Supplementary Table S3.

After dissection from the cadaver, TG specimens were fixed with 15% formaldehyde in 0.1 M phosphate buffer for 24 h at 4°C. Specimens were then dehydrated in sequential incubations of 70%, 80%, 95%, and 100% ethanol, followed by clearing with xylene as per a standard, pre-set ‘biopsy’ cycle in a tissue processor (ASP6025, Leica Biosystems) at RT under vacuum. Specimens were then embedded in paraffin wax and sectioned sagittally (10 μm) on a rotary microtome (Leica Biosystems, HI 2235). Sections were floated in a water bath set at 38°C (Leica Biosystems, HI1210), mounted individually on SuperFrost slides, and allowed to dry at RT for at least 18 h before storage at RT indefinitely.

### 2.9 Histology—Mouse and Rat

Rat and mouse histology was performed as previously described (Rees et al., 2021a; Ghanizada et al., 2021). Briefly, sections were thawed at RT, washed twice with TBS-T, then blocked with TBS-T containing 10% normal donkey serum (v/v) for 1 h at RT. Sections were then incubated with primary antibodies, diluted in TBS-T with 1% (v/v) normal donkey serum (immunobuffer), and incubated overnight at 4°C. For TG histology, sections were co-incubated with primary antibodies (pAb188, 1:100 (mouse) or 1:200 (rat); pAb36001, 1:500; β tubulin III, 1:500 or NF200, 1:200). Sections were then washed with TBS-T twice and incubated with secondary antibodies (1:200) in immunobuffer with DAPI for 1 h at RT. After secondary antibody incubation, sections were washed twice with TBS-T and coverslips mounted with ProLong Diamond Antifade (P36965, ThermoFisher Scientific).

Primary and secondary antibody details are outlined and compared in Supplementary Table S1. For all experiments, sections from each individual mouse or rat were processed separately in independent experiments. TG sections were obtained from the middle third of the ganglia in the sagittal plane. Sections were observed under a light microscope to check for morphology, tissue quality and neuron numbers. Sections displaying good tissue condition and sufficient neuron numbers (visually estimated to be > 200 neurons) were selected for staining. Triple staining of CGRP and CTR with β tubulin III or NF200 was performed in parallel on serial sections, generating two technical replicates of CGRP and CTR co-staining per animal. Sections were imaged using an Operetta high-content imaging system in non-confocal mode (rat pancreas) or confocal mode (mouse and rat TG) using a 20x high-numerical-aperture (0.75) objective (Perkin Elmer Life and Analytical Sciences, Waltham, MA). Some rat TG sections were also imaged with a 20x (0.8) lens on an LSM 710 laser scanning confocal microscope (Zeiss, Oberkochen, Germany).

### 2.10 Histology—Human

Postmortem human TG histology was performed as previously described (Ghanizada et al., 2021). Briefly, sections were heated at 60°C for 1 h, then dewaxed and rehydrated in xylene for 2 × 20 min, followed by 2 × 10 min of 100% ethanol, and 5 min each of 95%, 80%, and 75% ethanol. Sections were then washed for 3 × 5 min in water. Rehydration of tissue sections was performed at RT. Antigen retrieval was performed using 10 mM sodium citrate buffer, pH 6.0, at 121°C for 20 min. Sections were permeabilized with PBS + 0.2% Triton X-100 (v/v) (PBS-T) for 5 min, washed twice with PBS, then incubated with PBS containing 10% normal donkey serum for 1 h at RT. After blocking, sections were co-incubated with primary antibodies (mAb31-01, 1:250; pAb36001, 1:500; β tubulin III, 1:500 or S100, 1:200), diluted in PBS-T + 1% normal donkey serum (v/v) (human immunobuffer) overnight at 4^°^C. After primary antibody incubation, sections were washed once with PBS-T, twice with PBS, then incubated with secondary antibodies in human immunobuffer (1:250, Supplementary Table S1) for 3 h at RT. Sections were then washed with PBS, incubated with Hoechst (1:10,000) for 10 min at RT, washed again with PBS and the coverslips mounted with ProLong Diamond Antifade.

For all experiments, sections from each human case were processed separately as independent experiments. Sections were observed under a light microscope to check for morphology, tissue quality and neuron numbers. Sections displaying good tissue condition and sufficient neuron numbers (visually estimated to be > 150 neurons) were selected for staining. Triple staining of CGRP and CTR with β tubulin III or S100 was performed in parallel on serial sections, generating two technical replicates of CGRP and CTR co-staining per human case. Sections were imaged using an Operetta high-content imaging system in confocal mode using a 20x high-numerical-aperture (0.75) objective.

### 2.11 Immunoblotting (Western Blotting)

Preparation of whole cell or whole tissue lysates and western blotting were performed as previously described (Hendrikse et al., 2022). Transfected HEK293S cells in 15 cm^2^ dishes were washed with ice-cold PBS. The cells were harvested on ice in 10 ml of ice-cold PBS using a cell scraper. Cells were pelleted by centrifugation (1,000 x g, 10 min, 4^°^C) and the supernatant removed. One 15 cm^2^ dish corresponded to one whole cell lysate preparation. Transfected HEK293S cell pellets, fresh-frozen mouse (C57BL/6J), rat (SD) or human tissue were homogenized using a 1 ml Dounce glass homogenizer in immunoprecipitation buffer (Tris-NaCl, 0.1% SDS, 0.5% sodium deoxycholate, 1% Triton X-100, pH 8.0) containing a complete mini EDTA-free protease inhibitor cocktail tablet (Cat #4693159001, 1:10,000; Roche Applied Science). The homogenized samples were then left to solubilize for 2 h at 4^°^C. Samples were centrifuged (16,000 x g, 20 min, 4°C), and the supernatant was aliquoted into protein LoBind tubes and stored at -80°C. The protein concentration of a sample was quantified using a bicinchoninic acid protein assay kit (ThermoFisher Scientific).

Protein samples were incubated for 1 h at 37°C in 4x loading dye (2.5 ml 1 M Tris-HCL, 4 ml 20% sodium dodecyl sulfate, 4 ml 100% glycerol, 0.04 mg bromophenol blue) and 0.1 M DTT. Protein samples (0.1–20 µg, Supplementary Table S4) were loaded alongside the ab116027 (Abcam) or PrecisionPlus (BIORAD, 1610373) protein ladders onto 4–12% SurePage SDS gels (GenScript, Piscataway, NJ) and run at 180 V in MOPS buffer. Due to availability issues multiple protein ladders were used. The Abcam and PrecisionPlus ladders displayed comparable apparent molecular weights and were in line with each other (Supplementary Figure S1). Proteins were transferred to 0.45 µm PVDF (mouse/rat TG) or nitrocellulose membranes (human TG/TN) (Life Technologies and BIORAD) and were blocked with 5% low-fat milk in TBS-T for 1 h at RT. Blots were incubated with primary antibody (mAb8B9, 1:500; pAb188, 1:500; mAb31-01; 1:500) overnight at 4°C, washed twice with TBS-T, then incubated with secondary antibody (1:2,000, Supplementary Table S1) for 1 h at RT. Blots were washed twice with TBS-T, developed with Supersignal West Pico Plus ECL (ThermoFisher Scientific) and imaged using an Amersham Imager A600 (GE Healthcare). Image acquisition was performed using the automated exposure function with the high dynamic range setting. All blots presented are representative results from at least three independent experiments.

### 2.12 Image Preparation and Processing

Representative immunocytochemistry, western blotting and immunohistochemistry images are presented from at least three independent experiments performed using separate antibody dilutions. Independent immunocytochemistry experiments are defined as the immunoreactivity detected in cells from independent transfection and staining experiments performed with two technical replicates. Western blotting experiments are defined as independent experiments generated using one transfected cell lysate preparation or different tissue lysates prepared from three individual rodents and two human cases. Independent immunohistochemistry images are defined as the immunoreactivity detected in tissue from individual rodents or human cases.

Images were minimally processed using the FIJI open-source imaging platform to adjust color and brightness for presentation purposes (Schindelin et al., 2012). Any processing was uniformly applied across each image and all conditions for an antibody. Brightness and contrast, unless otherwise stated, were adjusted to the top and bottom of the histogram to allow visualization of staining across all intensities and prevent loss of data (Johnson, 2012). Therefore, minimally processed images are presented for most figures. However, for some figures, as noted in their legends, adjustment of contrast and brightness was made via the histogram (contrast stretching) to enhance visualization of positively stained cells with varying intensities. This did not affect the study conclusions.

### 2.13 Image Analysis and Statistical Analysis

To determine the size of neuronal cell bodies and quantify the proportion of stained neuronal cell bodies, image analysis was performed on multiple fields of view (20x high NA lens, Operetta) on each section from individual animals; three fields of view for mice and five for rats. Each field of view contained between ∼50–150 neurons. In total 1967 and 2205 neurons were analyzed for mouse and rat, respectively. All images were analyzed as unedited 16-bit TIFFs in greyscale. Image analysis was performed using FIJI and was partially automated using the macro function to generate counts and the mean diameter (mean of Feret diameter and minimum diameter for each neuron) of neurons demonstrating immunostaining for β tubulin III, CGRP, CTR and CTR/CGRP (see Supplementary Material: Supplementary method and Supplementary Figure S2A).

Graphing and statistical analysis was performed using Prism GraphPad 8.0.2 (GraphPad Software, La Jolla, CA). For each section/animal, neuronal cell body diameter data from all three or five fields of view were center binned in 2.5 µm increments for the four different neuron subsets (β tubulin III, CGRP, CTR and CTR/CGRP). These data were then plotted to generate histograms to visualize the combined data from each species, presented as mean ± standard error of the mean (s.e.m.) from six individual animals. A cubic spline was applied to help visualize the distribution.

The validity of the image and data analysis procedures were confirmed using staining of the pan neuronal marker β tubulin III (Supplementary Figure S2B). The neuronal cell body size distribution histograms generated for rat and mouse β tubulin III were consistent with the literature (Ambalavanar and Morris, 1992; Ruscheweyh et al., 2007; Lennerz et al., 2008). This indicated that the analysis was robust and analysis of the CGRP and CTR sub-populations within the total β tubulin III population was appropriate. NF200 immunoreactivity was not amenable to this analysis approach and therefore image quantification was restricted to β tubulin III.

For each section/animal, the proportion (percentage) of neurons expressing CGRP or CTR were determined for each of the fields of view. These were combined to give a mean percentage value for each animal. The mean percentage values were combined for each sex and species as appropriate and presented as mean ± s.e.m from three (sex) or six (species) individual animals. The same approach was used to assess the proportion of CTR + neurons which co-expressed CGRP. For statistical analysis, the combined mean values from three (sex) or six (species) individual animals were compared using Student’s t-tests. Statistical significance was defined as *p* < 0.05. Image analysis and quantification was not performed for human TG due to the lower number of human cases and variability in staining patterns between the different cases.

## 3 Results

### 3.1 Distribution of CTR and CGRP in Rat and Mouse TG

To examine the spatial relationships between CTR and CGRP, we first needed to identify and characterize an anti-CGRP antibody. Four anti-CGRP antibodies were tested (Supplementary Figure S3). In immunoblotting, all four anti-CGRP antibodies detected rat and human CGRP. Interestingly, immunoreactivity was more intense for rat, than for human CGRP (Supplementary Figure S3A). There was no cross-reactivity in immunoblotting with high amounts of amylin and no immunoreactivity in rat pancreatic islets for three of the CGRP antibodies, pAb36001, mAb81887 and pAbC8198 (Supplementary Figure S3A, C). However, mAbABS 026–05-02 displayed cross-reactivity with 100 µg of rat amylin in dot blots and immunoreactivity in rat pancreatic islets (Supplementary Figure S3A, C). All four anti-CGRP antibodies displayed similar patterns of immunoreactivity in rat TG neuronal cell bodies (Supplementary Figure S3D).

The primary anti-CGRP antibody pAb36001 was selected for further studies based on a combination of factors. It was able to detect CGRP with sufficient sensitivity in immunofluorescence and immunoblotting and did not cross-react with amylin under the conditions used (Supplementary Figure S3, Supplementary Table S5). Additionally, as pAb36001 was raised in goat it enabled colocalization with antibodies against CTR and other cellular markers, which were raised in rabbit or mouse. For CTR, we used pAb188 for these experiments. pAb188 has been knockout validated in several studies and displays robust immunoreactivity in several regions in rodent nervous tissue (Goda et al., 2018; Coester et al., 2020; Hendrikse et al., 2022).

To localize the CTR-like and CGRP-like immunoreactivity (LI) in the TG, sections were co-incubated with anti-CGRP, anti-CTR and primary antibodies for neuronal cell markers, β tubulin III (pan-neuronal) and NF200 (rodent A-fiber neuronal marker) (Shiers et al., 2020; von Buchholtz et al., 2020). For the purposes of stepwise description of the data, CGRP and CTR results are first presented individually with cellular markers, and then they are presented together to examine their spatial relationship.

#### 3.1.1 CGRP-like Immunoreactivity

**FIGURE 1:**
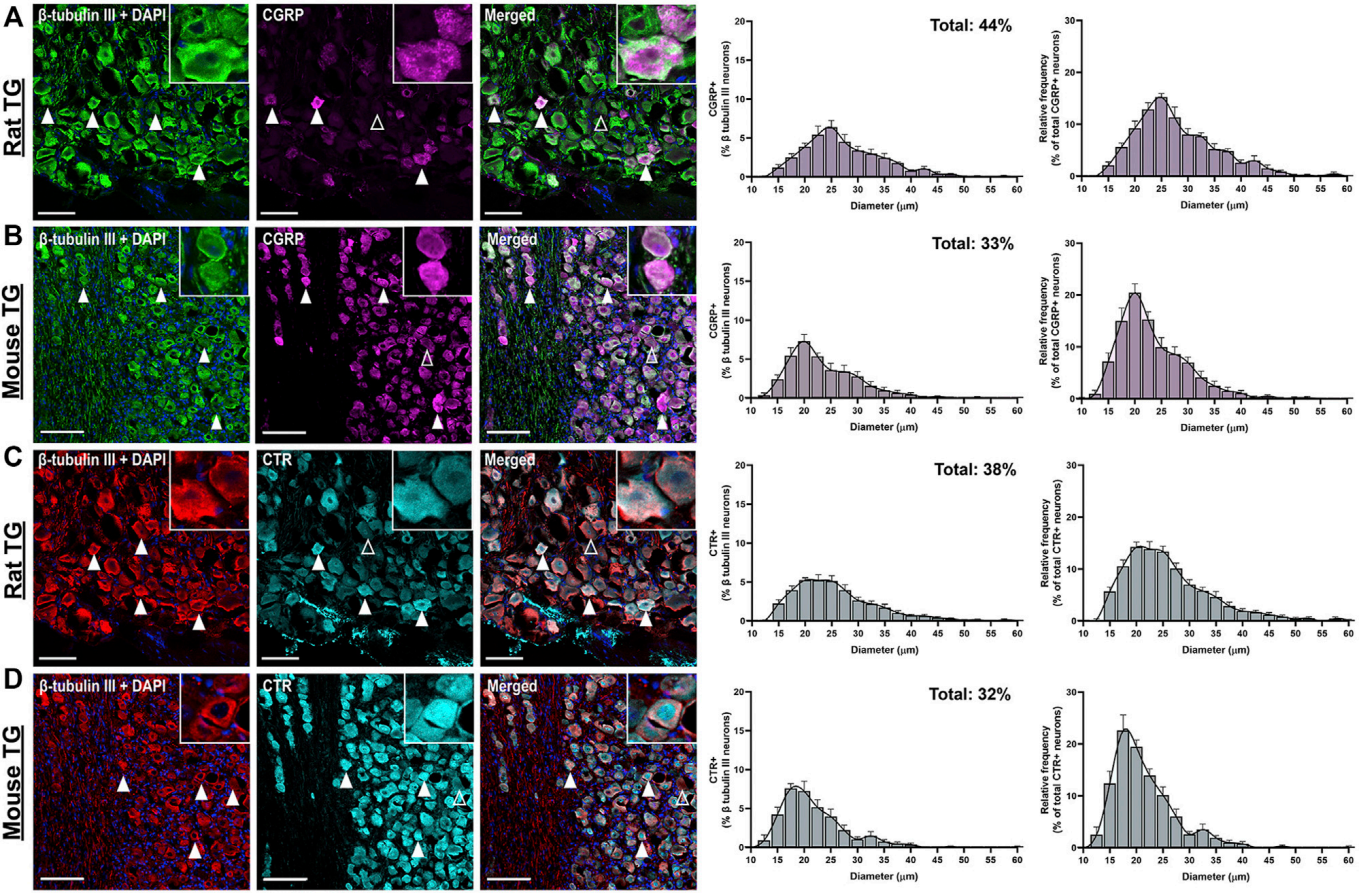
Immunohistochemical localization and quantification of CGRP (pAb36001, 10 µg/ml) or CTR (pAb188, 10 or 20 µg/ml) individually with β tubulin III (2 µg/ml) in adult rat and mouse TG. **(A)** CGRP and β tubulin III in rat **(B)** CGRP and β tubulin III in mouse **(C)** CTR and β tubulin III in rat and **(D)** CTR and β tubulin III in mouse. Filled white arrowheads indicate examples of positive staining; empty arrowheads indicate examples of an absence of staining. Image brightness and contrast were adjusted for presentation purposes and merged in FIJI. Scale bar = 100 μm. Images are representative of six rats and six mice (three male and three female). The size distribution (diameter) of neuronal cell bodies expressing CGRP or CTR for each species are displayed as histograms. The distribution of neuron size was quantified relative to the total β tubulin III (pan-neuronal marker) expressing neuron population and then relative to either CTR or CGRP expression. Data are the mean ± s.e.m, combined from six individual rats or mice (three male and three female).

CGRP-LI was present in the cell bodies of 44 ± 3.9% of rat and 33 ± 3.1% of mouse TG neurons. The size distribution was consistent with that of small to medium-sized neurons (Rat: 15–35 µm; Mouse: 10–30 µm), as indicated by β tubulin III staining (Figures 1A,B) (Messlinger and Russo, 2019). Immunoreactivity was usually observed as puncta in medium-sized neurons, indicating the expression of CGRP in vesicles, or dense/intense staining in smaller neurons (Figures 1A,B, Supplementary Figures S4–S5). No notable CGRP-LI was observed in satellite glia surrounding the neurons, nor the myelinating Schwann cells (Figures 1A,B). Visually, CGRP-LI appeared to be more frequent in mice than rats. However, this is likely due to lower signal intensity above background in mice, in combination with the limited histogram adjustment during image processing.

**FIGURE 2:**
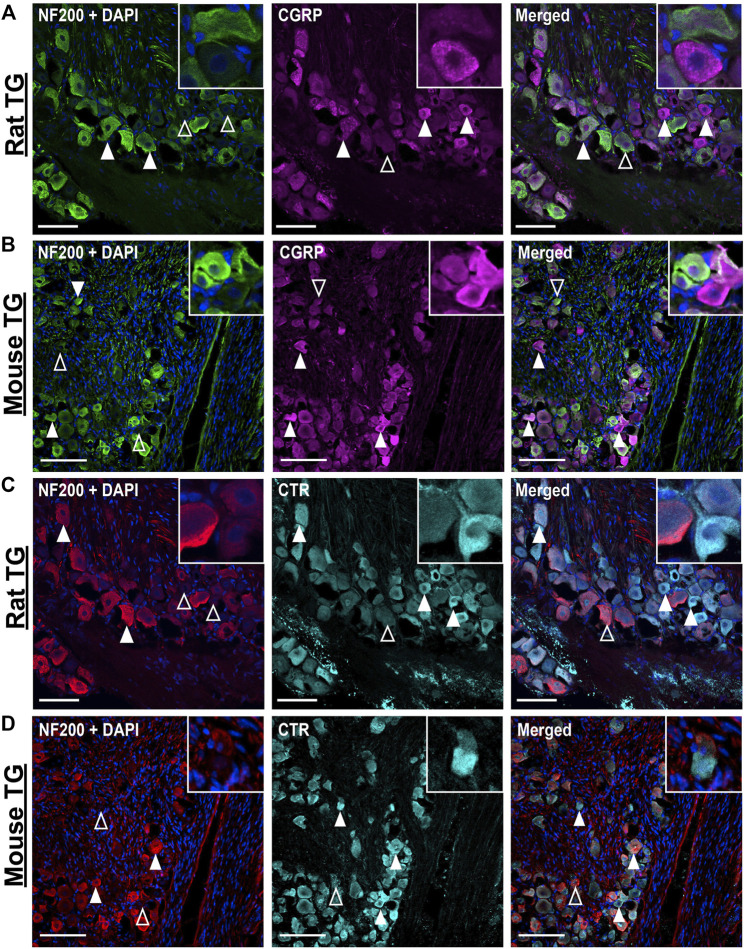
Immunohistochemical localization of CGRP (pAb36001, 10 µg/ml) or CTR (pAb188, 10 or 20 µg/ml) individually with NF200 (3 µg/ml) in adult rat and mouse TG. **(A)** CGRP and NF200 in rat **(B)** CGRP and NF200 in mouse **(C)** CTR and NF200 in rat and **(D)** CTR and NF200 in mouse. Filled white arrowheads indicate examples of positive staining; empty arrowheads indicate examples of an absence of staining. Image brightness and contrast were adjusted for presentation purposes and merged in FIJI. Scale bar = 100 μm. Images are representative of six rats and six mice (three male and three female).

CGRP-LI did not notably overlap with NF200 (Figures 2A,B). “Pearl-like” or varicose CGRP-LI was observed in neuronal fibers, as indicated by β tubulin III, but not those expressing NF200, suggesting that CGRP is more commonly expressed in unmyelinated C-fibers (Supplementary Figures S4–S5). These data are strongly in agreement with previous publications, which indicate that CGRP is expressed in approximately 30–50% of neurons (Lennerz et al., 2008; Eftekhari et al., 2010; Eftekhari et al., 2013; Edvinsson et al., 2019; Edvinsson L. et al., 2020; Guo et al., 2020).

#### 3.1.2 CTR-like Immunoreactivity

CTR-LI was observed in the cell bodies of 38 ± 4.1% of rat and 32 ± 3.8% of mouse TG neurons (Figures 1C,D). These CTR-positive neurons tended to be small to medium in size. The intensity of the CTR staining was variable, with some occasional bright cells, whereas other positively stained neurons were more moderate in intensity. CTR staining was often diffuse and present throughout the cytoplasm of the neurons, rather than clearly localized to the cell surface. CTR staining did not appear to commonly overlap with NF200 staining (Figures 2C,D). CTR-LI did not appear to overlap with either β tubulin III or NF200 stained neuronal fibers, nor was any CTR-LI observed in satellite glia or Schwann cells (Figures 1C,D). Like CGPR-LI, CTR-LI visually appeared to be more intense in mice than rats but this is again due to lower signal to background and limited image processing.

#### 3.1.3 Relative Distribution of CGRP-like and CTR-like Immunoreactivity

**FIGURE 3:**
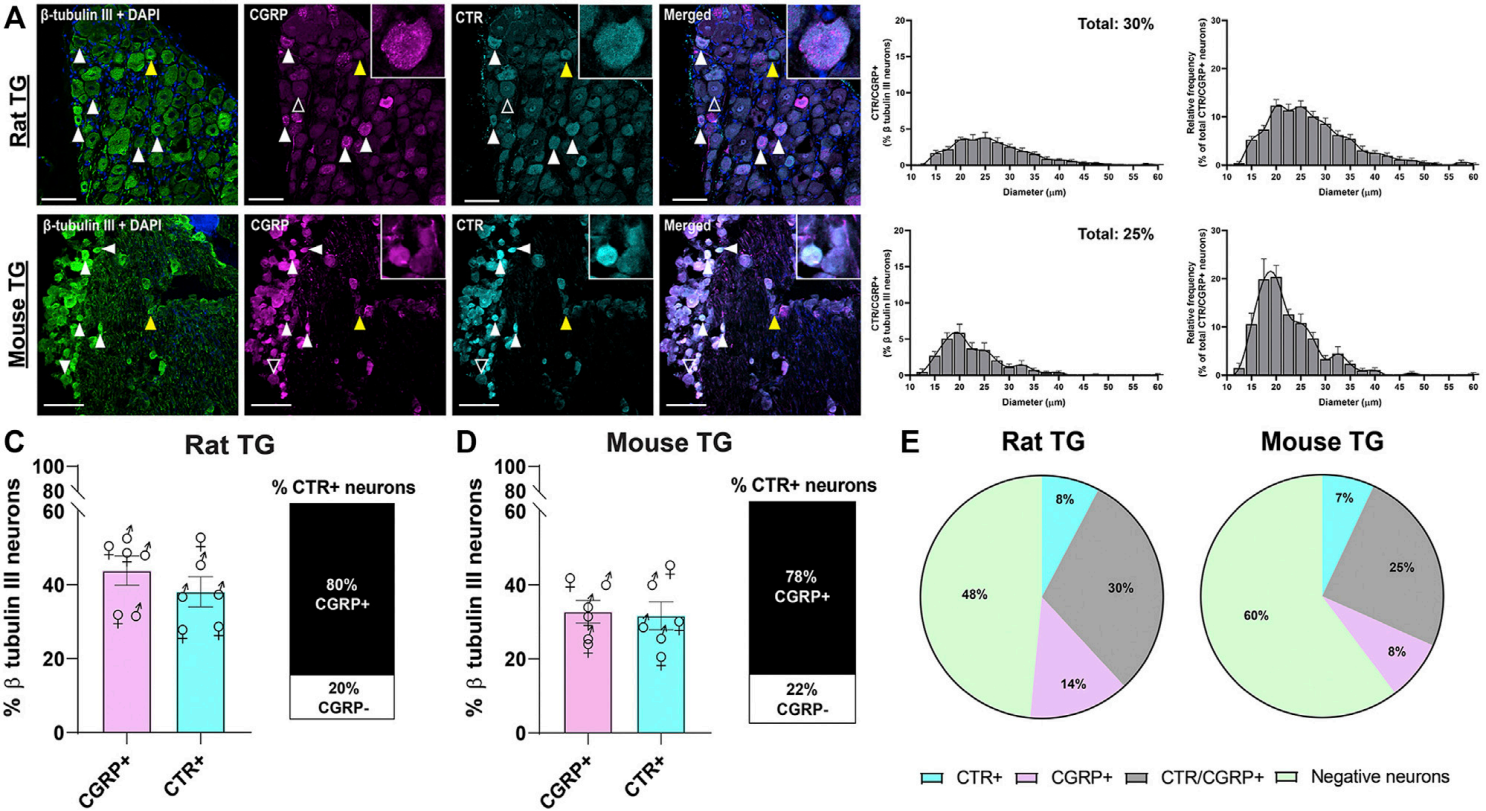
Immunohistochemical localization and quantification of CGRP (pAb36001, 10 µg/ml) and of CTR (pAb188, 10 or 20 µg/ml) together with β tubulin III (2 µg/ml) in adult rat and mouse TG. **(A)** CGRP and CTR in rat and **(B)** CGRP and CTR in mouse. β tubulin III is shown for reference. Filled white arrowheads indicate examples of positive staining; empty arrowheads indicate examples of an absence of staining; yellow arrowheads indicate expression in adjacent neurons. Image brightness and contrast were adjusted for presentation purposes and merged in FIJI. Scale bar = 100 μm. Images are representative of six rats or mice (three male and three female). The size distribution (diameter) of neuronal cell bodies expressing CGRP and CTR for each species are displayed as histograms. The distribution of neuron size was quantified relative to the total β tubulin III (pan-neuronal marker) expressing neuron population and then relative to CTR/CGRP expression. The percentage of **(C)** rat or **(D)** mouse β tubulin III TG neurons expressing CGRP or CTR and the proportion of CTR expressing neurons which also express CGRP TG. Data are the mean ± s.e.m, combined from six individual rats or mice (three male and three female). Percentage of the **(E)** total neuronal population (β tubulin III) which express CGRP alone without overlapping with CTR, CTR alone without overlapping with CGRP, or co-express CTR and CGRP in rat or mouse TG. Negative neurons refers to the neuronal population (β tubulin III) which do not express CGRP or CTR. Data are the mean, combined from six individual rats or mice (three male and three female).

**FIGURE 4:**
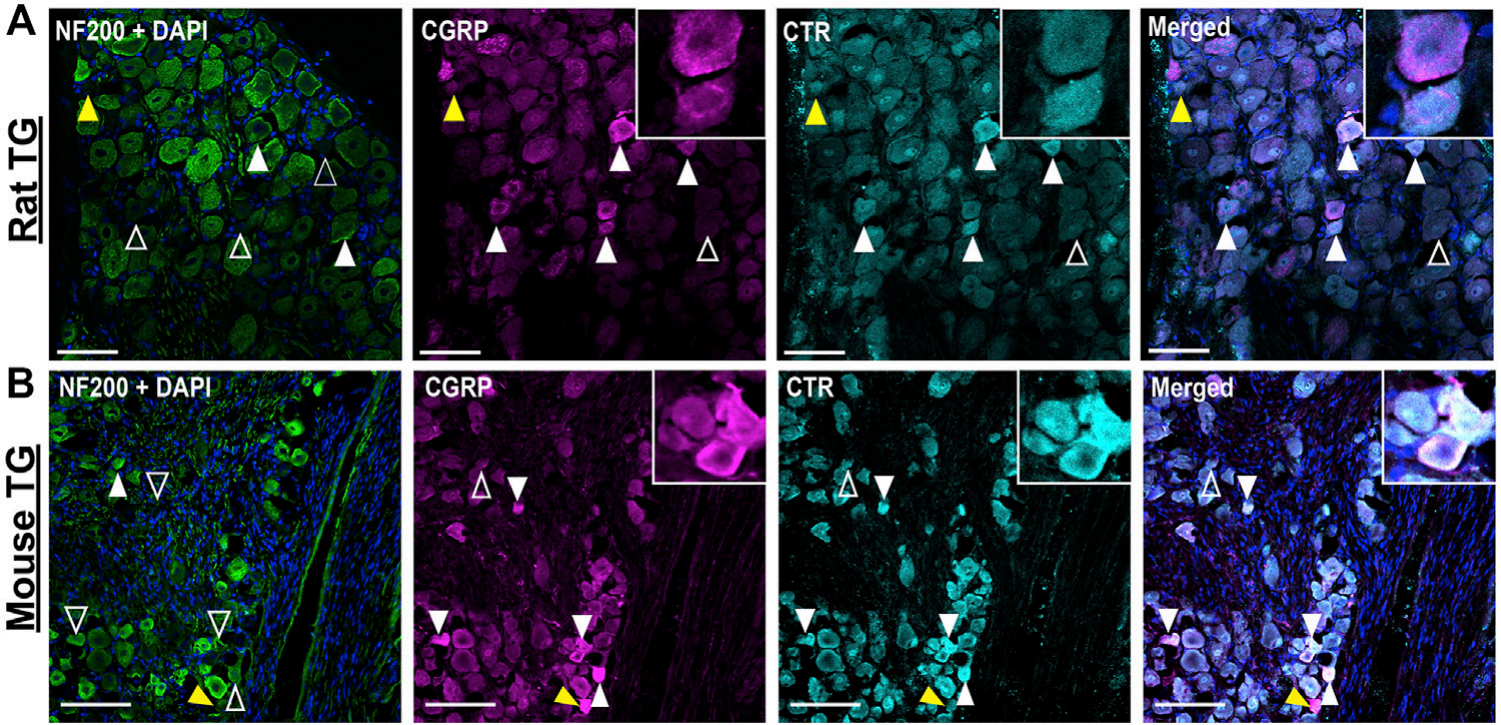
Immunohistochemical localization of CGRP (pAb36001, 10 µg/ml) and CTR (pAb188, 10 or 20 µg/ml) together with NF200 (3 µg/ml) in adult rat and mouse TG. **(A)** CGRP and CTR in rat and **(B)** CGRP and CTR in mouse. NF200 is shown for reference. Filled white arrowheads indicate examples of positive staining; empty arrowheads indicate examples of an absence of staining; yellow arrowheads indicate expression in adjacent neurons. Image brightness and contrast were adjusted for presentation purposes and merged in FIJI. Scale bar = 100 μm. Images and quantification are representative of six rats and six mice (three male and three female).

The distributions of CTR and CGRP-LI relative to one-another are shown in Figures 3, 4. CGRP-LI and CTR-LI was observed both in distinct and in overlapping populations of β tubulin III-positive neuronal cell bodies, indicating that there are neurons which express CGRP alone, CTR alone and co-express CGRP and CTR together. No notable co-staining of CTR and CGRP was observed in neuronal fibers, satellite glia or Schwann cells (Figures 3A,B).

Interestingly, 30 ± 4.3% and 25 ± 3.9% of all rat and mouse TG neurons, respectively, exhibited overlapping CGRP-LI and CTR-LI (white filled arrowheads), suggesting co-expression in these neurons. These neurons were typically small to medium in size (Figures 3A,B). The proportion of CTR-stained neurons that also exhibited CGRP-LI was 80 ± 4.6% of rat and 78 ± 1.7% of mouse TG neurons (Figures 3C,D).

Some neurons were positive for only CGRP or CTR (Figure 3E). In rat TG, the presence of neurons which exclusively expressed CGRP was more common than those which exclusively expressed CTR, with 13% of β tubulin III neurons expressing CGRP alone, compared to 8% for CTR alone. In mouse TG, exclusive expression of CGRP or CTR was 8 and 7%, respectively (Figure 3E). Neurons which displayed CTR-LI alone were often located next to neurons that expressed CGRP (yellow filled arrowheads). Co-staining of CTR and CGRP did not notably occur in NF200-expressing neurons (Figure 4).

There were no significant differences in the proportion of CGRP-LI or CTR-LI between rats and mice, nor the proportion of CTR-positive neurons which co-expressed CGRP (Table 1; Figures 3C,D). Similarly, there were no significant differences in CGRP-LI and CTR-LI, or CTR and CGRP co-expression between the female and male mice or rats (Table 2). The individual data points for each male or female rat or mouse are plotted in Figures 3C,D, to show the range and scatter of the data for each sex.

**TABLE 1:** Comparison of CGRP and CTR expression and CTR/CGRP co-expression between rat and mouse.

	CGRP+ (% β tubulin III neurons)	CTR+ (% β tubulin III neurons)	CGRP+ (% CTR+ neurons)
Rat	44 ± 3.9	38 ± 4.1	80 ± 4.6
Mouse	33 ± 3.1	32 ± 3.8	78 ± 1.7

Data are the mean ± s.e.m, combined from six individual rats or mice (three male and three female). Comparisons of the percentage of CGRP+ neurons, CTR+ neurons and the percentage of CTR+ neurons that co-express CGRP between rat and mouse were made by unpaired Student’s *t*-test. There were no significant differences.

**TABLE 2:** Comparison of CGRP and CTR expression and CTR/CGRP co-expression between female and male rat and mouse.

Species	Sex	CGRP+ (% β tubulin III neurons)	CTR+ (% β tubulin III neurons)	CGRP+ (% CTR+ neurons)
Rat	Female	43 ± 5.9	35 ± 8.2	74 ± 4.7
	Male	45 ± 6.4	41 ± 2.8	86 ± 7.0
Mouse	Female	31 ± 5.3	31 ± 7.2	79 ± 0.4
	Male	34 ± 4.1	33 ± 4.4	78 ± 3.8

Data are the mean ± s.e.m, combined from three individual female or male rats or mice. Comparisons between females and males of the percentage of CGRP+ neurons, CTR+ neurons and percentage of CTR+ neurons that co-express CGRP in rats or mice were made by unpaired Student’s *t*-test. There were no significant differences.

### 3.2 Immunoblotting - CTR Expression in Rat and Mouse TG

An orthogonal method, western blotting, was employed to gain additional molecular insight into CTR expression in rodent TG. The predicted molecular weight of the rat and mouse CT_(a)_ splice variant is approximately 52–53 kDa, although the CTR can also exist as other molecular forms due to alternative splicing, dimerization and post-translational modifications (Albrandt et al., 1995; Quiza et al., 1997; Harikumar et al., 2010). Rodent CTR protein expression was profiled using the monoclonal anti-rat CTR antibody mAb8B9, which has been well-validated and is suitable for western blotting (Hendrikse et al., 2022). In addition, the polyclonal anti-rat CTR antibody pAb188 was also used to allow a direct parallel to the immunohistochemistry data, although this antibody has been previously shown to be less suitable for western blotting (Hendrikse et al., 2022).

**FIGURE 5:**
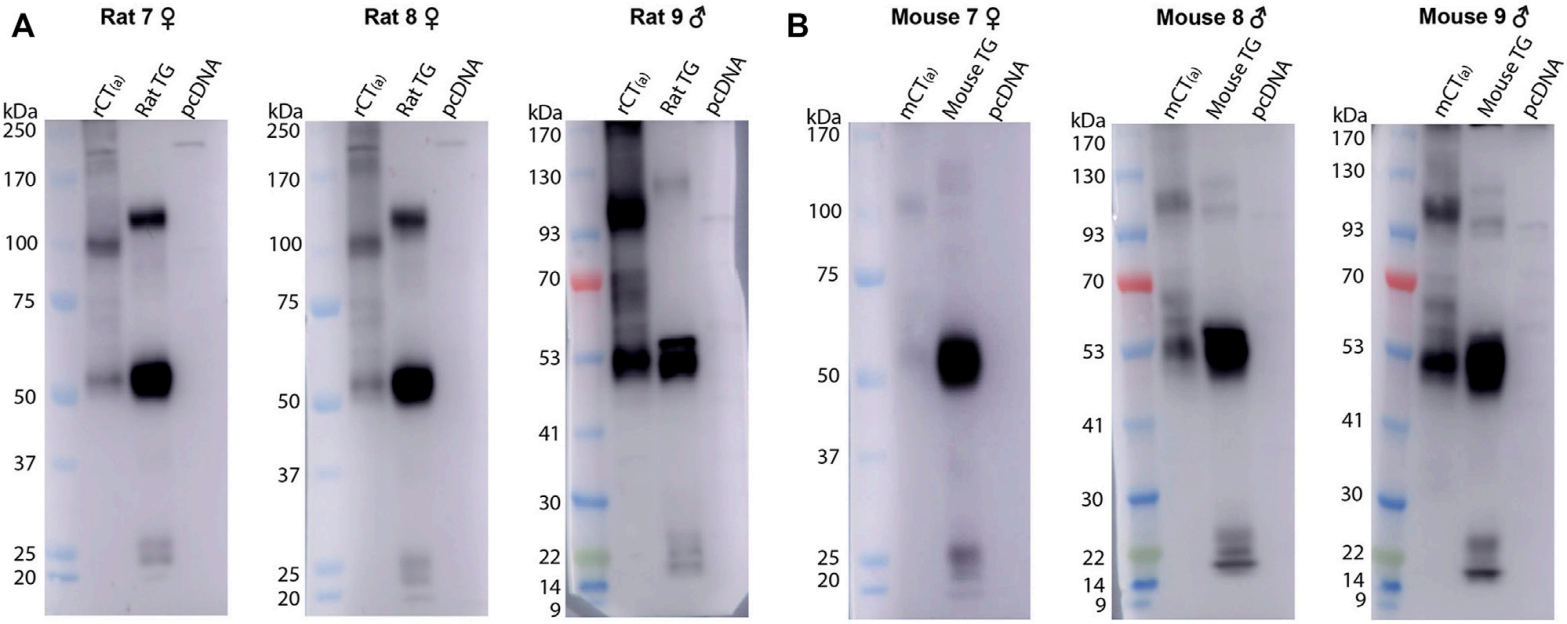
CTR-like immunoreactivity in rat and mouse TG lysate using immunoblotting with mAb8B9. **(A)** Immunoblots using lysate preparations from adult rat TG (20 µg) and HEK293S cells (10 µg) transfected with rat CT_(a)_ or vector alone (pcDNA). **(B)** Immunoblots using lysate preparations from adult mouse TG (20 µg) and HEK293S cells (10 µg) transfected with mouse CT_(a)_ or vector alone. Blots were probed with mAb8B9 (2 µg/ml). MW markers are shown on the left of each blot, with apparent MW in kDa. This image shows western blots of samples from individual mouse or rat TG. Images were adjusted uniformly for brightness and contrast for presentation purposes.

The mAb8B9 and pAb188 antibodies were both tested in the same tissue lysates from a mixture of male and female rodents. All blots for the mAb8B9 antibody are shown in Figure 5. In mCT_(a)_ and rCT_(a)_ transfected HEK293S cell lysates, mAb8B9 detected a clear immunoreactive band at ∼53 kDa and a weaker band at ∼100 kDa (Figure 5). Additionally, an immunoreactive smear from ∼65-70 kDa was also commonly observed. In rat and mouse TG lysate, a strong immunoreactive band was observed at ∼53 and ∼120 kDa with an additional band at ∼100 kDa present in mouse TG lysate, but not rat. Three lower molecular weight bands of less than 30 kDa were also observed in both species. Interestingly, a band at ∼56 kDa was observed in male but not in female rat and mouse TG lysate (Figure 5). Too few animals of each sex were used for any definitive conclusions to be made but this observation is reported so that future studies could be designed to formally test this. Additionally, the effect of estrous cycle on expression could be considered.

**FIGURE 6:**
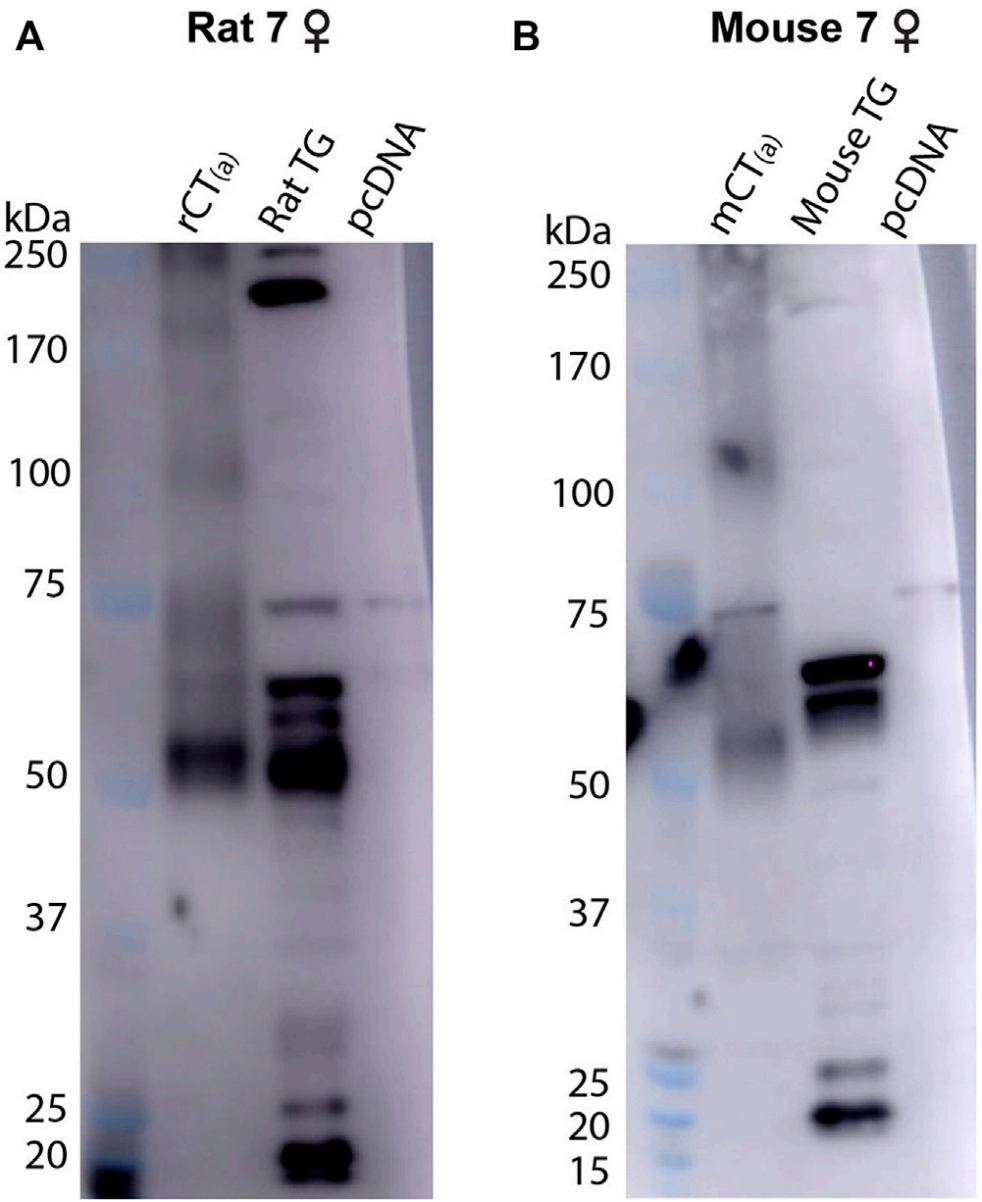
CTR-like immunoreactivity in rat and mouse TG lysate using immunoblotting with pAb188. **(A)** Immunoblots using lysate preparations from adult rat TG (20 µg) and HEK293S cells (0.1 µg) transfected with rat CT_(a)_ or vector alone (pcDNA). **(B)** Immunoblots using lysate preparations from adult mouse TG (20 µg) and HEK293S cells (0.3 µg) transfected with mouse CT_(a)_ or vector alone. Blots were probed with pAb188 (4 µg/ml). MW markers are shown on the left of each blot, with apparent MW in kDa. This image is representative of three western blots using TG lysate from three individual mice or rats (mixed sex) displaying results from a female **(A)** rat and **(B)** mouse. Images were adjusted uniformly for brightness and contrast for presentation purposes.

For pAb188, the band pattern was consistent between sexes and blots are representative of both sexes (Figure 6). In the mCT_(a)_ and rCT_(a)_ transfected HEK293S cell lysates, pAb188 detected an immunoreactive band at ∼52 kDa and a weaker band at ∼110 kDa. Additionally, an immunoreactive smear from 52–70 kDa was also commonly observed (Figure 6). Generally, pAb188 displayed similar immunoreactivity patterns to mAb8B9 in transfected cells, rat TG and mouse TG lysates. However, a greater number of non-specific bands were observed for pAb188, likely due to its polyclonal nature (Figure 6). The bands between 100 and 120 kDa seen for mAb8B9 were absent in rat and mouse TG lysates (Figure 6). Similarly, the wide, strong band at ∼52 kDa was absent in mouse TG lysate. Several additional strongly immunoreactive bands were observed at approximately 60 and 230 kDa. However, the ∼60 kDa band was also present in the vector-transfected cell lysate when a higher amount of protein was loaded, suggesting that it could be non-specific (Supplementary Figure S6, Supplementary Table S4).

Collectively, the data with pAb188 and mAb8B9 indicate that protein is present in rat and mouse TG at molecular weights consistent with that expected for CTR. Interestingly, the band sizes were not always directly comparable to the transfected cell samples.

### 3.3 Validation of Anti-Human CTR Antibodies

**FIGURE 7:**
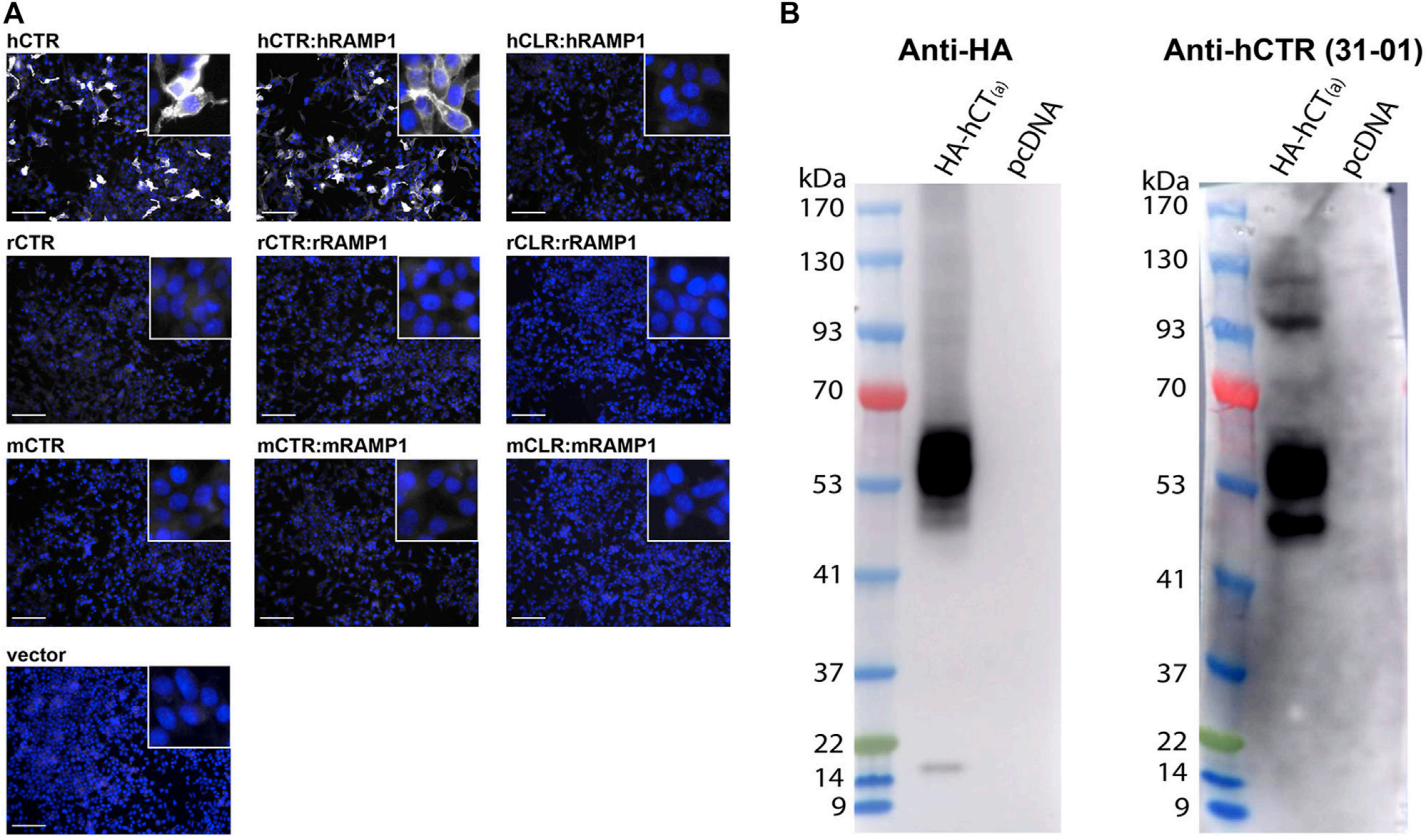
Validation of the anti-human CTR mAb31-01 antibody in transfected HEK293S cells. **(A)** Immunocytochemistry, with immunoreactive staining in greyscale, and nuclear DAPI staining in blue. Scale bar = 100 μm h, human; r, rat; m, mouse. Images are representative of three independent experiments in duplicate wells. **(B)** Immunoblots using cell lysate (10 µg) preparations from HEK293S cells transfected with HA-tagged human CTR or vector alone (pcDNA). Lanes were loaded with 10 μg of protein. Blots were probed with anti-HA (1 µg/ml) or mAb31-01 (2 µg/ml). MW markers are shown on the left of each blot, with apparent MW in kDa. This image is representative of four technical replicates using the same lysate preparations. The brightness and contrast of these images have been enhanced uniformly for presentation purposes.

We next proceeded to test human samples. To examine the expression of CTR in human TG the anti-human CTR antibody mAb31-01 was selected as it has been reported to detect human CTR in immunohistochemistry and immunoblotting, whereas our recent results suggest that pAb188 is not suitable for detecting human CTR (Wookey et al., 2008; Wookey et al., 2012; Ostrovskaya et al., 2019; Hendrikse et al., 2022). The mAb31-01 antibody was validated using immunocytochemistry and immunoblotting (Figure 7). The immunocytochemistry controls are provided in Supplementary Figure S7. The predicted molecular weight of non-glycosylated human CT_(a)_ is approximately 52 kDa (Nygaard et al., 1997; Quiza et al., 1997; UniProt, 2021).

In immunocytochemistry, mAb31-01 displayed strong immunoreactivity in cells transfected with human CTR, in the presence and absence of RAMP1 but little to no immunoreactivity in the vector control (Figure 7A). There was no visible detection of rat or mouse CTR or apparent cross-reactivity with CLR for any of the species tested. In immunoblotting, mAb31-01 displayed immunoreactivity at ∼50 kDa and a wider intense band at ∼53–63 kDa, similar to the HA control (Figure 7B). This is consistent with its ability to detect human CTR using immunoblotting in the existing literature (Wookey et al., 2012;Furness S. et al., 2016;Furness S. G. B. et al., 2016). Two bands at ∼100 and 120 kDa were also observed.

An additional two antibodies reported in the literature or by their commercial supplier to detect human CTR were also tested in immunocytochemistry in transfected cells to determine their potential suitability for studying CTR expression (Fu et al., 2017). pAbPA1-84457 displayed diffuse background staining and was unable to robustly detect human CTR, whereas pAb230500 could detect human CTR, but had a lower signal compared to background than mAb31-01 (Supplementary Figures S8–S9). Therefore, based on these results in transfected cells, we used mAb31-01 for subsequent experiments due to its strong detection of human CTR, low background staining, and its previous use in human tissue or primary cell lines.

### 3.4 Expression of CTR and CGRP in Human TG

To localize CTR-LI and CGRP-LI in human TG, sections were co-incubated with anti-CGRP pAb36001 (as rat and mouse studies), anti-CTR mAb31-01 and anti-β tubulin III antibodies. NF200 could not be used as a marker for A-fiber neurons as it is not discriminatory for these neuronal subpopulations in humans (Shiers et al., 2020). Pilot results indicated that CTR-LI and CGRP-LI may be present in glia, therefore, co-staining with S100, a glial marker, was also performed. CGRP and CTR results are initially described individually to allow comparisons with the cellular markers, then together to examine their spatial relationship.

#### 3.4.1 CGRP-like Immunoreactivity

**FIGURE 8:**
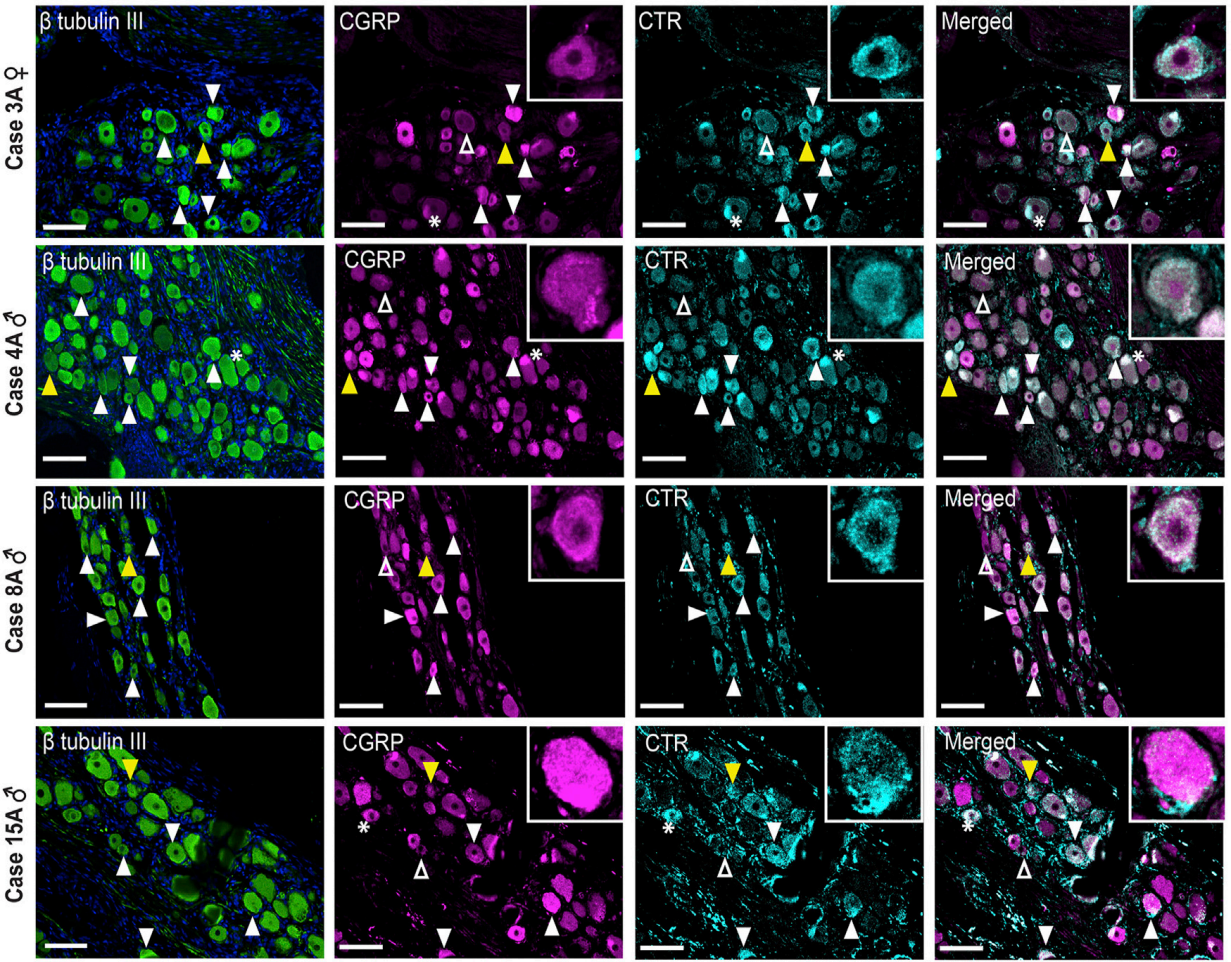
Immunohistochemical localization of CGRP (pAb36001, 10 µg/ml) and CTR (mAb31-01, 4 µg/ml) together with β tubulin III (1.2 µg/ml) in human TG. CGRP and CTR in four human cases (3A, 4A, 8A and 15A). β tubulin III is shown for reference. Filled white arrowheads indicate examples of positive staining; empty arrowheads indicate examples of an absence of staining; yellow arrowheads indicate expression in adjacent neurons. *Indicates examples of autofluorescence due to lipofuscin. Image brightness and contrast were adjusted for presentation purposes and merged in FIJI. Scale bar = 100 μm. Images are from each of the four human cases.

CGRP-LI was observed in the cell bodies of small to medium-sized neurons and as varicose fibers, overlapping with β tubulin III (Figure 8, Supplementary Figure S10). CGRP-LI was diffuse in the soma of smaller to medium-sized neuronal cell bodies or as puncta, indicating presence in vesicles (Figure 8; Supplementary Figure S10). Qualitative assessment of β tubulin III neuronal size suggests that CGRP-LI neurons are likely C-fiber neurons. Overlapping CGRP-LI and S100-LI was observed for some fibers in all four human cases. This suggests that either a subset of neuronal fibers co-express CGRP and S100 or that a subset of CGRP expressing neuronal fibers is encased by S100 expressing Schwann cells (Supplementary Figure S10). CGRP-LI was not observed in the satellite glia surrounding neuronal cell bodies for three of the four human cases, however, overlap of S100, and CGRP was observed in case 15A (Supplementary Figure S11). Overall, CGRP staining was relatively consistent across the human cases and agreed with previous studies (Del Fiacco et al., 1991; Eftekhari et al., 2010; Ghanizada et al., 2021).

#### 3.4.2 CTR-like Immunoreactivity

CTR-LI was present but was variable across the human cases (Figure 8). In case 3A, a female, intense CTR-LI was observed primarily in smaller neurons with more diffuse and less frequent staining in medium neurons. For cases 4, 8A, males, staining was more commonly observed in medium neurons than smaller neurons. A similar pattern was observed for case 15A, male, however staining of what appeared to be satellite glia was also observed (Supplementary Figure S11). No CTR-LI was observed in the neuronal fibers for any of these human cases.

#### 3.4.3 The Relative Distribution of CGRP-like and CTR-like Immunoreactivity

Image analysis and quantification was not possible due to the inherent variation in staining patterns between different human cases. However, apparent colocalization of CTR-LI and CGRP-LI was observed in neuronal cell bodies for all four human cases (Figure 8). Neurons that expressed CTR but not CGRP were sometimes located next to neurons that expressed CGRP. CGRP-LI together with CTR-LI followed the pattern of CTR staining, where colocalization was mostly seen in smaller neurons of case 3A and medium neurons for case 4A, 8A, 15A (Figure 8). Additionally, colocalization of CTR and CGRP staining was observed in the probable satellite glia of case 15A (Supplementary Figure S11).

### 3.5 Immunoblotting - CTR Expression in Human TG and TN

**FIGURE 9:**
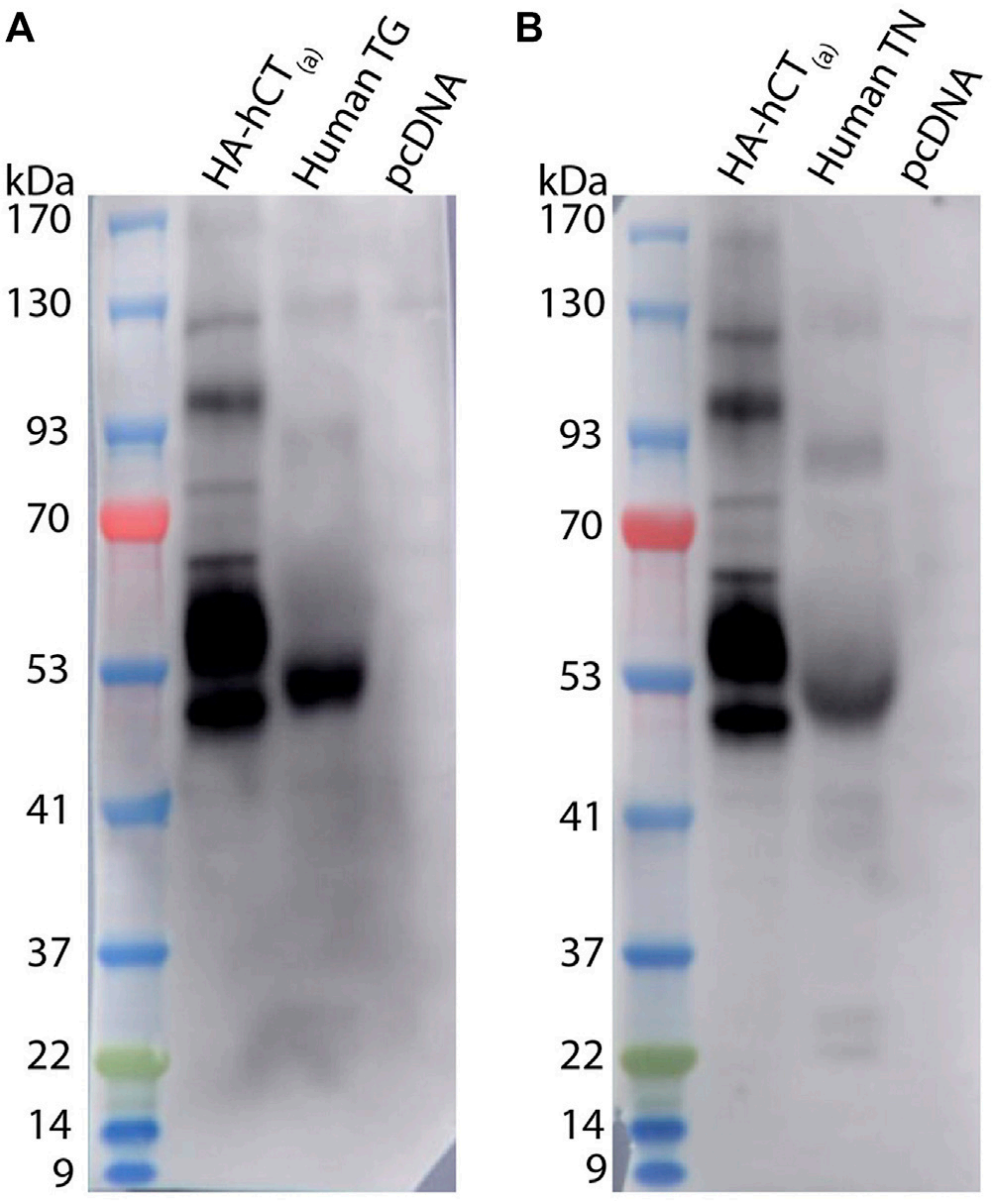
CTR-like immunoreactivity in human TG and TN lysate using immunoblotting. Immunoblots using lysate preparations from human TG and TN (20 µg) or HEK293S cells (10 µg) transfected with HA-tagged human CT_(a)_ or vector alone (pcDNA). Blots were probed with mAb31-01 (2 µg/ml), comparing immunoreactivity in **(A)** TG (case 1322) and **(B)** TN (case 1543). MW markers are shown on the left of each blot, with apparent MW in kDa. This image is from one western blot for two human cases. Images were adjusted uniformly for brightness and contrast for presentation purposes.

Western blotting (with mAb31-01) was also used as an orthogonal method to explore CTR expression in human samples. In human TG and TN, bands were observed at ∼52, 90 and 130 kDa, with the 52 kDa band having the most intense immunoreactivity of the three (Figure 9). Between the 22 and 37 kDa markers, two additional bands were present in the TN but not the TG lysate (Figure 9). Interestingly, the immunoreactive bands in the human TG and TN lysates did not directly overlap with the bands in the hCT_(a)_ control lysate. A potential explanation for this is splice variation, which is discussed below.

### 3.6 CGRP-like and CTR-like Immunoreactivity Distribution Across Species

**FIGURE 10:**
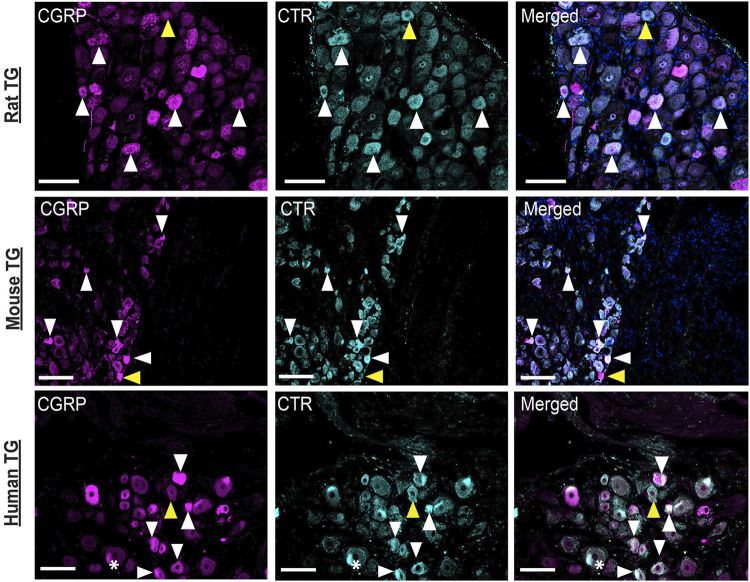
Comparison of CGRP-like and CTR-like immunoreactivity across species. CGRP and CTR in rat, mouse and human TG. White arrowheads indicate colocalization; yellow arrowheads indicate expression in adjacent neurons. *Indicates examples of autofluorescence due to lipofuscin. Image brightness and contrast were adjusted for presentation purposes and merged in FIJI. Additional histogram adjustment (contrast stretching) was performed for these images to convey the frequency and variation in the intensity of the immunoreactivity. Scale bar = 100 μm. Images are representative of six rats and mice (three male and three female). Human case 3A was used for this comparison.

Images of CGRP-LI and CTR-LI across species are collated in Figure 10 to enable comparison of staining patterns in the TG of rats, mice and humans. These images underwent additional adjustment of contrast and brightness via the histogram (contrast stretching) to enable greater visualization of positively stained cells of varying intensities, in line with acceptable image processing guidelines (Johnson, 2012).

Figure 10 shows that staining in all three species is similar, with some cells displaying intense immunoreactivity and others with more moderate immunoreactivity, suggesting varying levels of protein expression. Colocalization of CTR-LI and CGRP-LI in neuronal cell bodies, with occasional immunoreactivity in adjacent neurons, was observed for all three species. Overall, the distribution and colocalization of CGRP-LI and CTR-LI appear to be consistent in the TG of rats, mice and humans.

## 4 Discussion

### 4.1 CTR Is Expressed in the Cell Bodies of TG Neurons

In this study, CTR-LI was observed in the TG of three species when examined by immunohistochemistry and western blotting. This extends previous work which detected CTR in the TG of humans and rats and is the first report of CTR expression in mouse TG (Walker et al., 2015; Edvinsson L. et al., 2020). Additionally, this the first report quantifying the proportion of TG neurons that express CTR and provides the novel finding that CGRP may be co-expressed with CTR in individual neurons.

The distribution of CTR-LI in the cell bodies of small to medium-sized TG neurons is consistent with previous reports in rats and humans (Walker et al., 2015; Edvinsson L. et al., 2020). The CTR-LI distribution was similar in mouse TG. The size of neuronal cell bodies in the TG is known to be loosely correlated with the different neuronal subpopulations (Messlinger and Russo, 2019). The size of the CTR-LI neurons, in combination with the less frequent colocalization with NF200, suggests that CTR is expressed predominantly in C-fiber neurons in the TG (Ruscheweyh et al., 2007; Shiers et al., 2020). These data provide further evidence supporting the expression of CTR in the TG, however, the relative distribution across the whole TG remains unknown and should be considered in future studies. CTR-LI was not present in all cells, potentially explaining why studies examining CTR mRNA expression have given variable results (Barrett et al., 2013; Manteniotis et al., 2013; Flegel et al., 2015; Walker et al., 2015; LaPaglia et al., 2018).

The pattern of CTR expression was relatively consistent between individual rats and mice, however, there were differences between human cases, particularly in relation to the size of neurons that contained CTR-LI. A greater variation is not unexpected in the human samples. The case history of migraine in these individuals is unknown. Recently, it was reported that for some migraineurs an amylin agonist, pramlintide, but not CGRP, was able to induce migraine-like attacks (Ghanizada et al., 2021). It is possible that for these patients an amylin receptor, such as the AMY_1_ receptor, may make a greater contribution to migraine genesis. Populations of neurons which express amylin-responsive receptors could underlie this sensitivity. However, the sample size in this study was small and further research is required. Future studies should also investigate the presence of RAMP1 and other RAMPs, together with CTR (Walker et al., 2015; Hendrikse et al., 2019). This relies on the availability of antibodies that pass rigorous validation tests. It is also possible that CTR without RAMPs has a role to play in the TG because administration of calcitonin and activation of CTR alone has previously been shown to be anti-nociceptive, including in migraine sufferers (Micieli et al., 1988; Ustdal et al., 1989; Humble, 2011; Ito et al., 2012). It is important to note that in interpreting the presence of CTR in the TG, that CTR pharmacology differs between species with mouse CTR being more responsive to CGRP, especially βCGRP, than human and rat CTR (Garelja et al., 2021a).

In western blotting, the apparent molecular weight of the bands was consistent with the predicted molecular weight of CTR and previous reports (Quiza et al., 1997; Tikellis et al., 2003; Wookey et al., 2012; UniProt, 2021). This also indicated the presence of multiple molecular forms of CTR in the TG, which could be due to splice variants, dimers, post-translational modifications or translation and degradation products (Gorn et al., 1992; Anusaksathien et al., 2001; Seck et al., 2003; Harikumar et al., 2010; Gilabert-Oriol et al., 2017). For example, the size of some of the bands in the TG lysates suggests that the insert positive CT_(b)_ variant, which is approximately 2–5 kDa larger than CT_(a)_ in humans and rodents, may also be present in addition to the CT_(a)_ variant (Albrandt et al., 1993; Moore et al., 1995; Quiza et al., 1997). In addition to several distinct bands which could correspond to specific splice variants, immunoreactivity at approximately 50–52 kDa was often observed as a wide band. This suggests that there may be multiple CTR forms of similar molecular weights, such as CT_(a)_ and CT_(b)_, present in the TG. The observation of potential CTR variants in the trigeminovascular system provides an avenue for future investigation because different CTR splice variants have different pharmacological and cell signalling profiles (Houssami et al., 1994; Moore et al., 1995; Qi et al., 2013; Yu et al., 2013; Wnorowski and Jozwiak, 2014; Dal Maso et al., 2018). Interestingly, some differences in immunoreactivity were observed for mAb8B9 and pAb188 in rat and mouse TG lysates which may be due to their monoclonal and polyclonal nature (Lipman et al., 2005).

### 4.2 CTR and CGRP Colocalize in C-Fiber Neurons

The expression of CGRP was consistent with prior reports. CTR and CGRP colocalized in the cell bodies of several neurons or were observed in adjacent neurons. A similar pattern of CGRP-LI and CTR-LI was evident in mouse, rat and human TG. This cross-species consistency provides support for a conserved mechanism being at play in the function of these proteins. Notably, a similar distribution of CTR-LI was observed in all species, even though a distinct antibody was used in the human samples. Colocalization tended to occur in small to medium sized neurons, suggesting that colocalization occurs in C-fiber neurons (Messlinger and Russo, 2019). This was supported by qualitative assessment indicating that colocalization did not tend to occur in NF200 (an A-fiber marker) expressing neurons (Ruscheweyh et al., 2007). Additionally, single cell mRNA expression data from a TG atlas indicates that CGRP and CTR mRNA are both present in non-A-fiber, peptidergic and non-peptidergic nociceptor neurons in the mouse TG (Yang et al., 2022).

This contrasts with previous studies examining the relative distribution of CLR-LI and CGRP-LI, which consistently report that these are present in distinct neuronal populations (Lennerz et al., 2008; Eftekhari et al., 2010; Eftekhari et al., 2013; Edvinsson et al., 2019). The presence of CTR-LI in or nearby to cells with CGRP-LI suggests that CGRP could act as the local agonist for CTR-based receptors, such as the AMY_1_ receptor, in the TG. This implies that CTR-based receptors could mediate some aspects of CGRP function in the TG. It is also possible that other CTR agonists, calcitonin and amylin, could act via this receptor in the TG. However, the source of these agonists is more likely to be systemic than locally produced because the TG is not a notable source of these peptides, compared to that of CGRP (Rosenfeld et al., 1983; Goadsby et al., 1990; Manteniotis et al., 2013; Flegel et al., 2015; Irimia et al., 2020; Rees et al., 2021b; Ghanizada et al., 2021).

The presence of CGRP either in the same neurons as CTR or in neurons close in proximity to CTR-expressing neurons supports the potential involvement of a CTR-based receptor in pain modulation. Their size is consistent with C-fiber neurons, which are reported to have a greater contribution to the long-term potentiation of pain and sensitization in migraine than A-fiber neurons (Henrich et al., 2015; Levy et al., 2019). In particular, the activation of meningeal and dural afferent C-fibers, which project from the TG, have been linked to sensitization (Bartsch and Goadsby, 2003). Additionally, CGRP may possibly promote cortical spreading depression, which has been linked to the development of migraine and its symptoms, via both C- and A-fiber neurons (Charles and Baca, 2013; Close et al., 2019; Filiz et al., 2019).

### 4.3 A CTR-Based Receptor Could Underlie CGRP’s Autoregulatory Action

Autocrine regulation of CGRP in the TG has been speculated for many years (Messlinger et al., 2020). Several studies have reported that genes upregulated by CGRP can, in turn, upregulate CGRP itself (Durham and Russo, 2003; Bellamy et al., 2006; Durham, 2006; Zhang et al., 2007). Furthermore, gold labelled-CGRP appeared to bind to neurons that expressed CGRP and stimulation of TG neuronal cultures with CGRP increases CGRP mRNA (Segond von Banchet et al., 2002; Zhang et al., 2007). Interestingly, in an animal model of chronic migraine, mice chronically treated with nitroglycerine (NTG) have increased CGRP expression in the TG (Iversen and Olesen, 1996; Dieterle et al., 2011; Pradhan et al., 2014; Dallel et al., 2018). TG neuron cultures from this mouse model were also more responsive to CGRP than those treated with vehicle (Guo et al., 2020). However, chronic NTG treatment did not increase the number of neurons expressing CGRP. This suggests that the upregulation of CGRP mRNA previously observed may be primarily within neurons which already express CGRP (Messlinger et al., 2012). Additionally, this increased responsiveness to CGRP appeared to primarily be regulated via C-fiber neurons. How this apparent autoregulation of CGRP is mediated has remained unanswered as CLR and CGRP are not reported to commonly colocalize in TG neurons (Lennerz et al., 2008; Messlinger et al., 2020). It has therefore been previously speculated that co-expression of CTR and CGRP might occur (Guo et al., 2020; Messlinger et al., 2020). Our data indicating co-expression of CTR and CGRP in C-fiber neurons support the hypothesis that a CTR-based receptor, with a strong candidate being the AMY_1_ receptor, could mediate CGRP upregulation and response in the TG (Guo et al., 2020).

Differences in the degree of autocrine signaling could occur between individual migraine patients, where enhanced autocrine signaling of CGRP in TG neurons could account for some patients who are unresponsive to anti-CGRP antibody therapy. Although this is speculative, it is based on estimates that significantly more anti-ligand antibody, in the realm of four to eight orders of magnitude greater than the peptide-receptor binding affinity, is required to inhibit autocrine signaling (Forsten and Lauffenburger, 1992a). In these cases, a receptor-targeted approach would be preferable (Forsten and Lauffenburger, 1992b). Currently, erenumab is the only approved anti-receptor antibody for the prevention of migraine, which targets the CGRP receptor, but does some have some ability to block the AMY_1_ receptor (Garelja et al., 2021b; Bhakta et al., 2021). Erenumab can potently block CGRP receptor activation of A-fiber neurons to inhibit firing (Melo-Carrillo et al., 2017). However, erenumab does not appear to lower the firing rate of activated C-fiber neurons in response to CGRP (Melo-Carrillo et al., 2017). This raises the question of whether erenumab is sufficient to inhibit the potential autocrine activation of CTR-based receptors, such as the AMY_1_ receptor, in C-fiber neurons. The gepants should also be considered for their potential effects, given that they also have affinity for the AMY_1_ receptor (Garelja et al., 2021b).

## 5 Conclusion

In this study, we identified that CTR and CGRP can be co-expressed in TG neurons. This may underpin the reported autocrine actions of CGRP, which have been linked to migraine chronification. Additionally, multiple molecular forms of CTR were present in TG, indicating that multiple pharmacologically unique receptors could contribute to craniofacial pain. Although current migraine treatments which target CGRP and the canonical CGRP receptor provide relief for many migraine patients, there are patients who do not respond, suggesting that there is scope to develop further treatments (Edvinsson et al., 2018; Dubowchik et al., 2020). CTR-based receptors, such as the AMY_1_ receptor, have been proposed as potential targets (Walker et al., 2015; Irimia et al., 2020; Ghanizada et al., 2021). Our study supports the hypothesis that such receptors may contribute to migraine and highlights the importance of developing specific antibodies or antagonists targeting CTR-based receptors, such as the AMY_1_-receptor, to probe the drug development potential of this receptor in the treatment or prevention of migraine.

## Data Availability

The raw data supporting the conclusion of this article will be made available by the authors, without undue reservation.
