# Supplementary material for: CGRP and the Calcitonin Receptor are Co-Expressed in Mouse, Rat and Human Trigeminal Ganglia Neurons
Source: Front Physiol. 2022 May 10;13:860037. doi: 10.3389/fphys.2022.860037 (PMC9128745; doi:10.3389/fphys.2022.860037)
Supplement: Supplementary file 1 [file DataSheet1.pdf]

Supplementary Material

1 Supplementary methods

Image analysis using FIJI:

The image analysis workflow is summarized in Supplementary Figure 2. Prior to analysis of the images the minimum threshold for CGRP and CTR staining was determined for each rat or mouse. Due to the variability in staining patterns and intensity (e.g. for CGRP this ranged from intense cytoplasmic staining to puncta), global thresholding could not be applied for CGRP and CTR. Therefore, a minimum threshold was determined for each animal by analyzing the fluorescence intensity of 15-20 positive and negative neurons in two fields of view for each rat or mouse. The intensity of the positive and negative neurons were statistically summarized (descriptive statistics) using Prism GraphPad 8.0.2 and the 10th percentile value of the positive staining was used as the minimum threshold for that particular rat or mouse in the image analysis. To confirm that this value would not result in the inclusion of non-specific background staining this was compared to the 90th percentile intensity value for negative neurons. In all cases the 10th percentile value of positive neurons and 90th percentile value of negative neurons did not overlap, suggesting that any staining selected for by this thresholding method would likely be capturing positive staining above background.

Images were then analyzed using a FIJI macro. First, the β tubulin III image was thresholded using the triangle global threshold, set to automatic, and a binary mask created. The shape filter plugin was applied (area: 0.0003, fill holes) to remove some of the fiber staining. The neuronal cell bodies were then separated by adjustable watershed (1.5) and another shape filter was used (area: 0.0003, elongation: 0-0.8) to remove debris and fiber staining. CGRP and CTR images were analyzed next by thresholding with the predetermined appropriate minimum threshold for each animal. These images were similarly converted to binary masks and underwent a shape filter (area: 0.0003, fill holes), adjustable watershed and another shape filter (area: 0.0003, elongation 0-0.8) to remove debris, non-specific speckles and fiber staining. To overlay the β tubulin III mask with the CGRP or CTR masks, morphological reconstruction was used (MorphoLib plugin). The β tubulin III/CGRP and β tubulin III/CTR masks were then overlayed with MorphoLib to generate a CGRP/CTR mask to show neurons which only co-express CGRP and CTR. The properties of the three overlayed masks were then adjusted with the correct pixel width/height for the 20x high NA Operetta lens. The area and Feret diameter were determined for each individual neuron using "Analyze Particles" and were also summarized with counts for β tubulin III neurons, CGRP+ neurons, CTR+ neurons, and CTR/CGRP+ neurons. During method development, summarized counts and diameters were compared to manual counting and measurement for a subset of animals as a quality control and validation of the analysis.

2 Supplementary Data

2.1 Figures

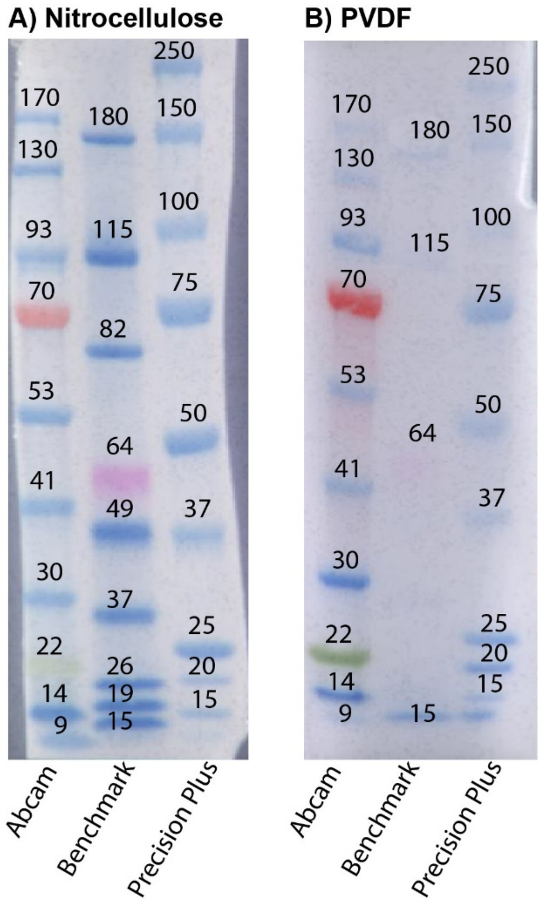

Supplementary Figure 1. Comparison of ladders and their provided molecular weights on nitrocellulose and PVDF membrane. Images are from one experiment. Apparent molecular weights (kDa) are labelled above the bands. Previous analysis of the Precision Plus ladder indicates that the apparent molecular weights run accurately under these conditions (Neris et al., 2020).

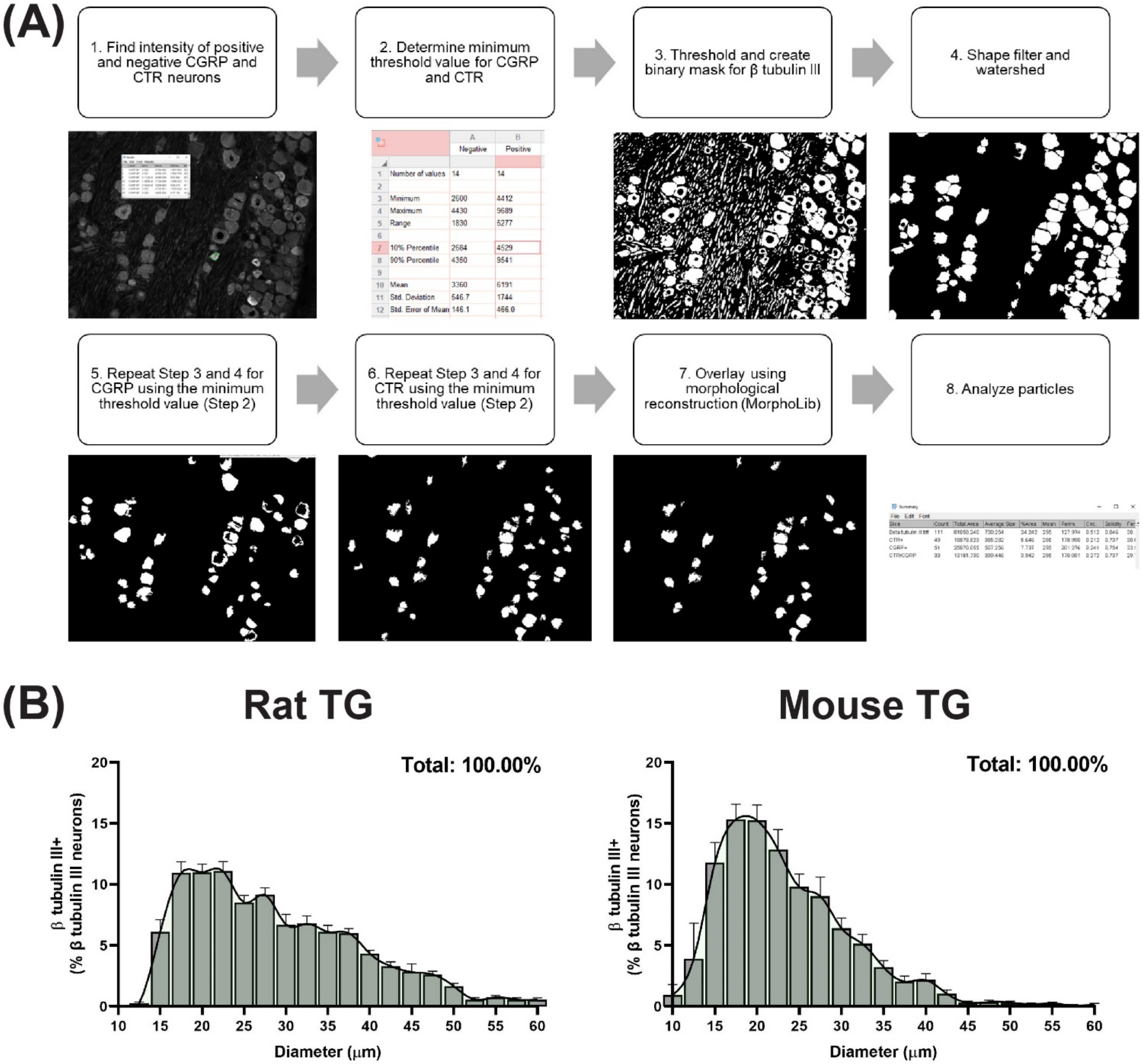

Supplementary Figure 2. Workflow of image analysis and size distribution (diameter) of neuronal cell bodies expressing the pan-neuronal marker β tubulin III in rat and mouse TG. (A) Image analysis workflow using Prism and FIJI. Example images are from rat TG. (B) The distribution of neuronal size was analyzed relative to the total neuronal population for rats and mice. Data are the mean ± s.e.m, combined from six individual rats or mice (three male and three female).

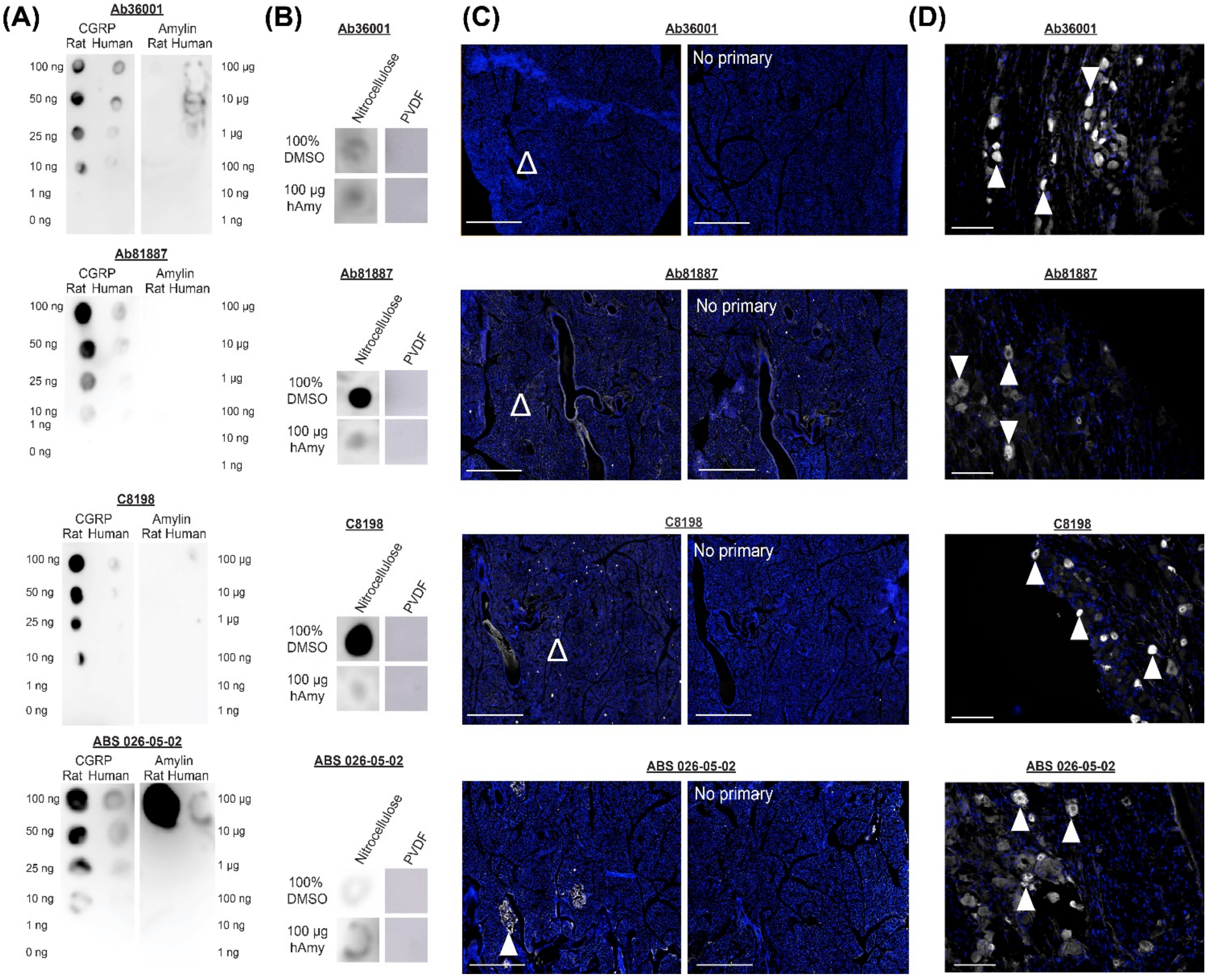

Supplementary Figure 3. Characterization of four anti-CGRP antibodies. (A) Dot blots testing specificity using human and rat CGRP and amylin on nitrocellulose membrane. The image is representative of 3-5 independent experiments. (B) Dot blots comparing the detection of 100 µg of human amylin (hAmy) dissolved in 100% DMSO and 100% DMSO alone on nitrocellulose and PVDF membrane. All four CGRP antibodies appear to interact with DMSO at varying levels on nitrocellulose membrane. Conversely, neither non-specific interactions with DMSO, nor cross-reactivity with 100 µg of hAmy were observed on PVDF membrane, suggesting that an interaction between the antibodies, DMSO and the nitrocellulose membrane is causing non-specific immunoreactivity. The images are representative of 1-3 independent experiments. (C) Immunoreactive staining (Ab36001, 10 µg/ml; Ab81887, 10 µg/ml; C9198, 1:4000; ABS 026-05-02, 2.65 µg/ml) in rat pancreatic islets and no primary control to further examine cross-reactivity in a tissue which highly expresses amylin. Empty arrowheads indicate examples of unstained islets and the filled arrowhead indicates an example of a stained islet. Scale bar = 500 µm. The brightness and contrast of these images was uniformly enhanced for presentation purposes. (D) CGRP-LI in rat TG, scale bar = 100 µm. The filled arrowheads indicate examples of positive neuronal staining. Antibody staining in greyscale and DAPI staining of nuclei in blue. Images are representative of three immunohistochemical experiments performed in three rats.

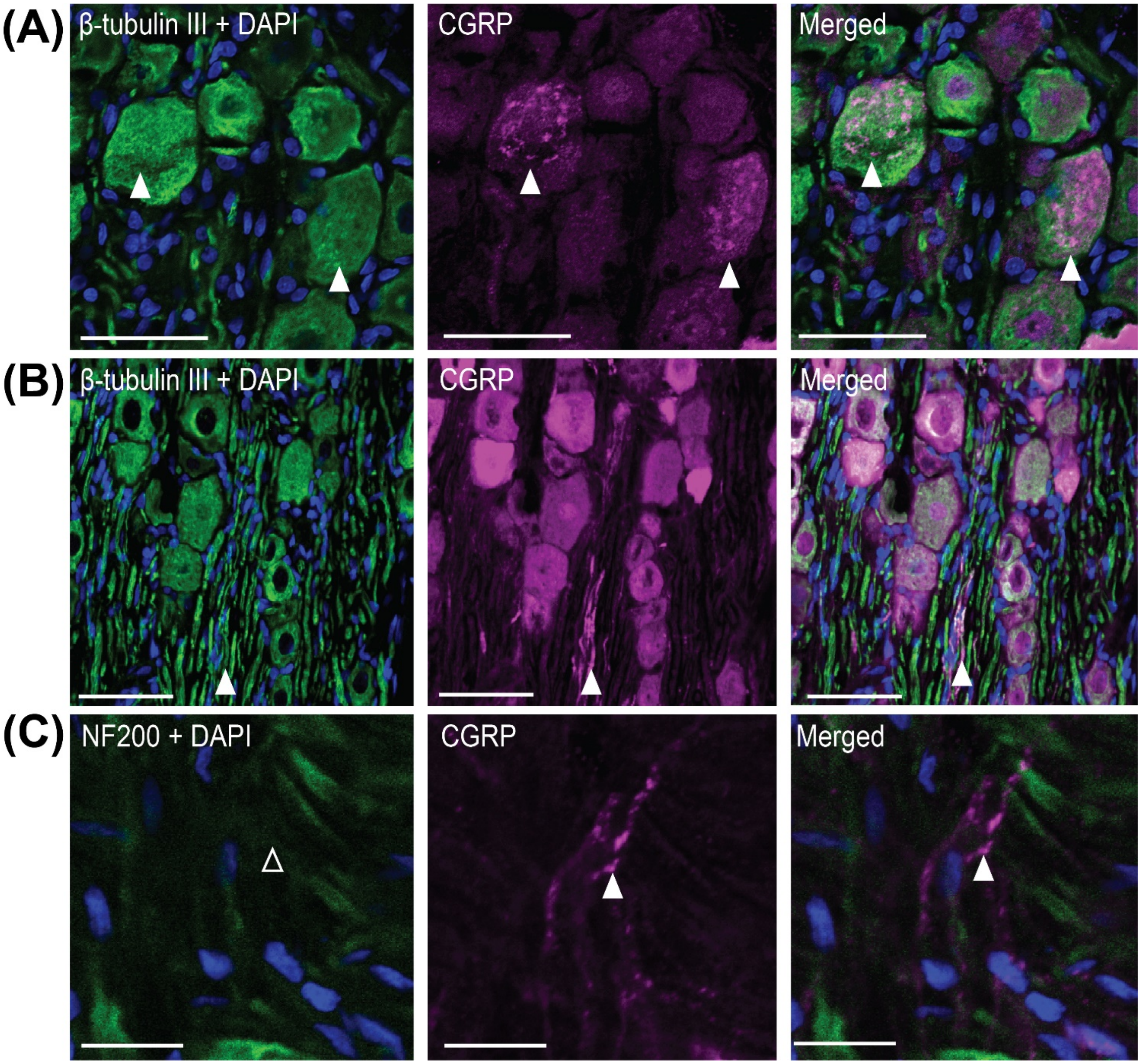

Supplementary Figure 4. Immunohistochemical localization of CGRP (Ab36001, 10 µg/ml) with β-tubulin III (2 µg/ml) and NF200 (3 µg/ml) in adult rat TG. Distribution of CGRP-like immunoreactivity (LI) (A) in puncta or (B, C) in fibers, relative to β tubulin III or NF200. Filled arrowheads indicate examples of positive staining; empty arrowheads indicate examples of an absence of staining. Scale bar = (A, B) 50 or (C) 25 µm. Image brightness and contrast were adjusted (contrast stretching) for presentation purposes and merged in FIJI. Images are representative of six rats (three male and three female).

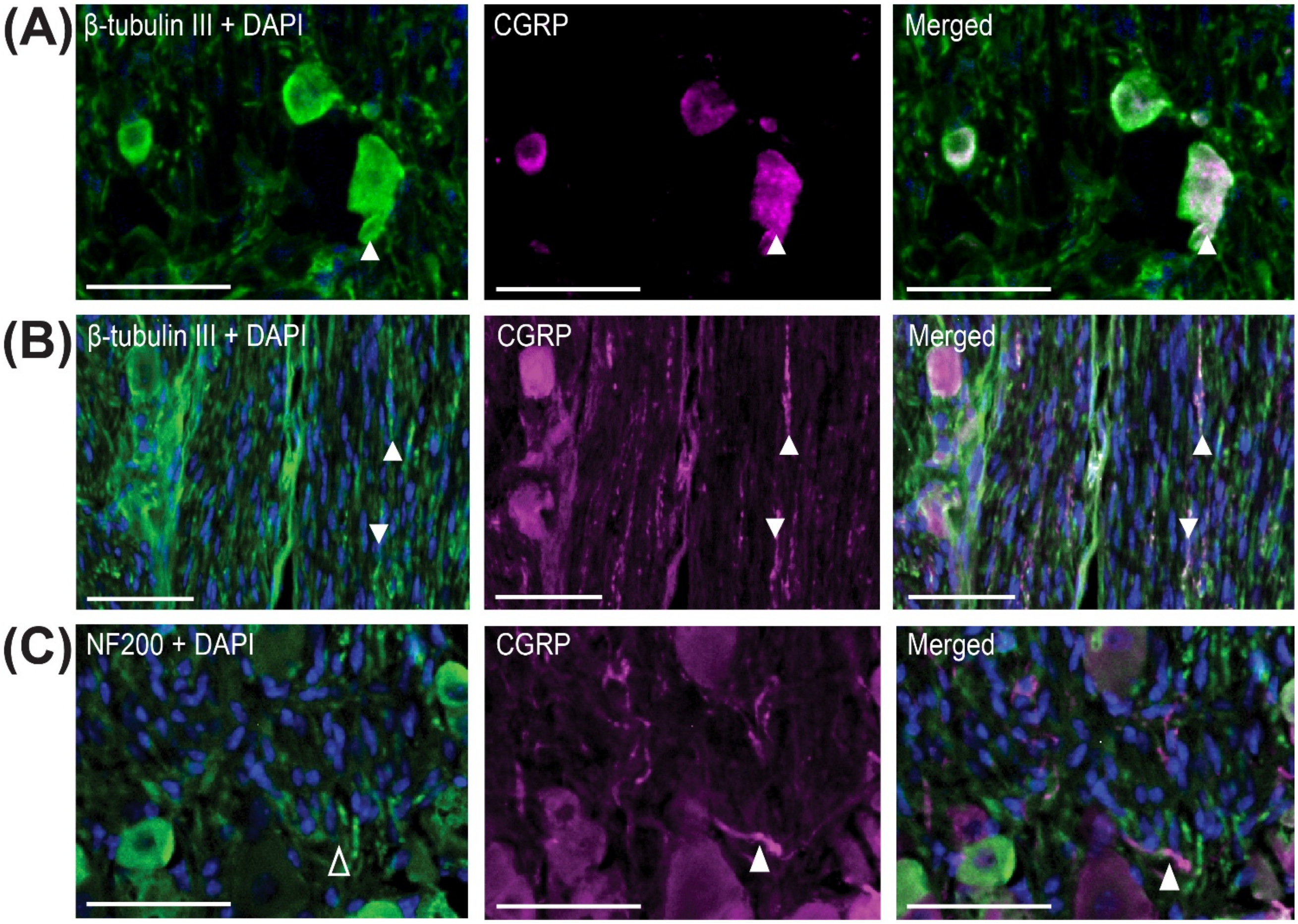

Supplementary Figure 5. Immunohistochemical localization of CGRP (Ab36001, 10 µg/ml) with β-tubulin III (2 µg/ml) and NF200 (3 µg/ml) in adult mouse TG. Distribution of CGRP-LI (A) in puncta or (B, C) in fibers, relative to β tubulin III or NF200. Filled arrowheads indicate examples of positive staining; empty arrowheads indicate examples of an absence of staining. Scale bar = 50 µm. Image brightness and contrast were adjusted (contrast stretching) for presentation purposes and merged in FIJI. Images are representative of six mice (three male and three female).

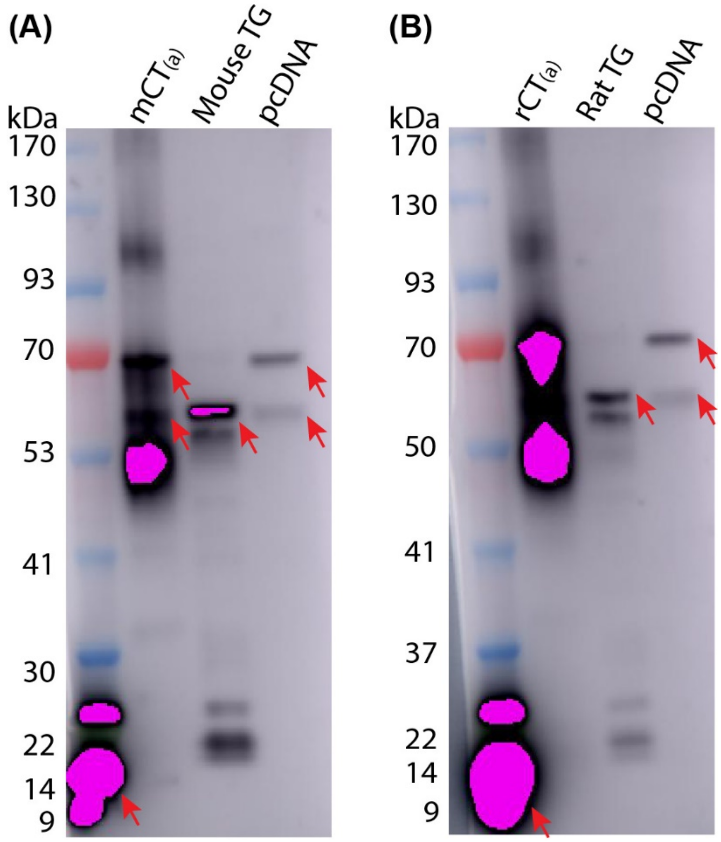

Supplementary Figure 6. Additional non-specific pAb188 bands. (A) Immunoblots using lysate preparations from adult mouse TG (20 µg) and HEK293S cells (10 µg) transfected with mouse $CT_{(a)}$ or vector alone (pcDNA). (B) Immunoblots using lysate preparations from adult rat TG (20 µg) and HEK293S cells (10 µg) transfected with rat $CT_{(a)}$ or vector alone. Blots probed with pAb188 (4 µg/ml). Red arrows indicate likely non-specific bands which appear in the vector control sample or the ladder. MW markers are shown on the left of the blots, with apparent MW in kDa. Pink indicates overexposure. This image is representative of two western blots using individual mice or rats (one male and one female). Images were adjusted uniformly for brightness and contrast for presentation purposes.

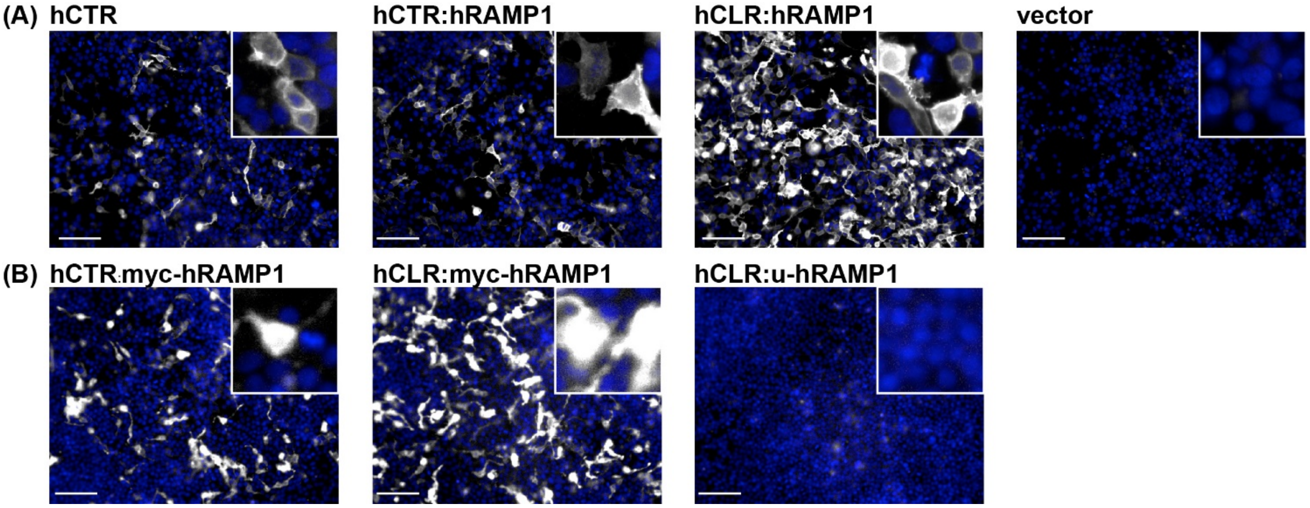

Supplementary Figure 7. Immunocytochemical staining of transfected HEK293S cells using anti-HA and anti-myc antibodies. (A) anti-HA (1 µg/ml) or (B) anti-myc (0.8 µg/ml). Immunoreactive staining is shown in greyscale and nuclear DAPI as blue. h = human; u, untagged. Scale bar = 100 µm. Images are representative of two to eight independent experiments in duplicate wells. For (A) and (B) brightness and contrast of these images have been enhanced uniformly within the image set for presentation purposes.

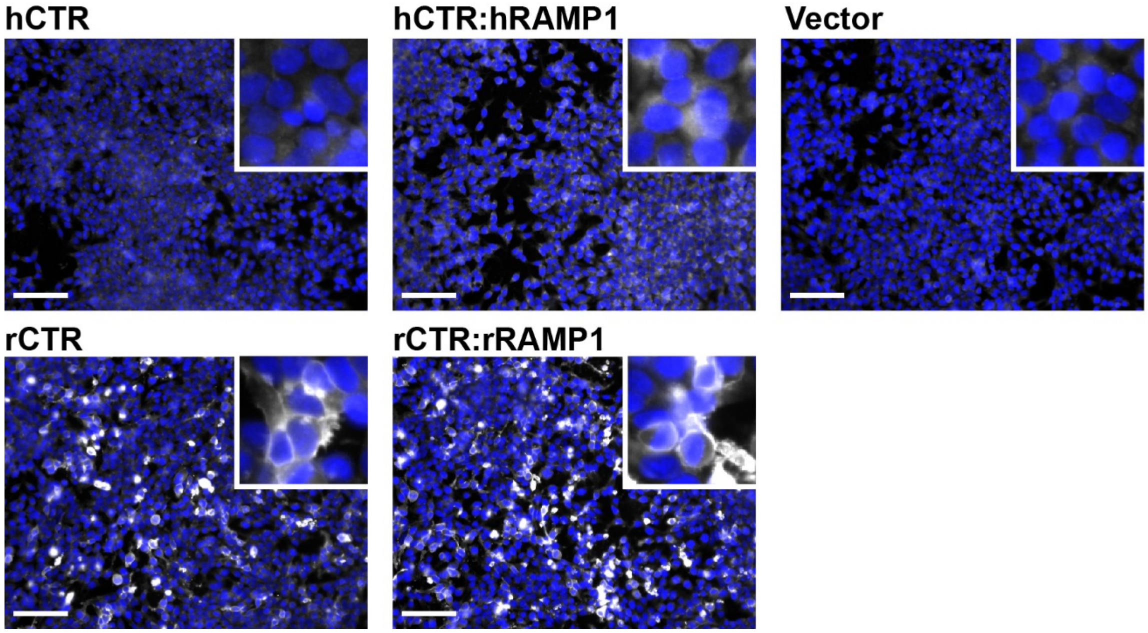

Supplementary Figure 8. Immunocytochemical staining of transfected HEK293S cells using the anti-CTR pAbPA1-84457 antibody (10 µg/ml). Immunoreactive staining is shown in greyscale, and nuclear DAPI staining is shown in blue. h = human; r = rat. Scale bar = 100 µm. Images are representative of three independent experiments in duplicate wells. The brightness and contrast of these images has been enhanced uniformly for presentation purposes.

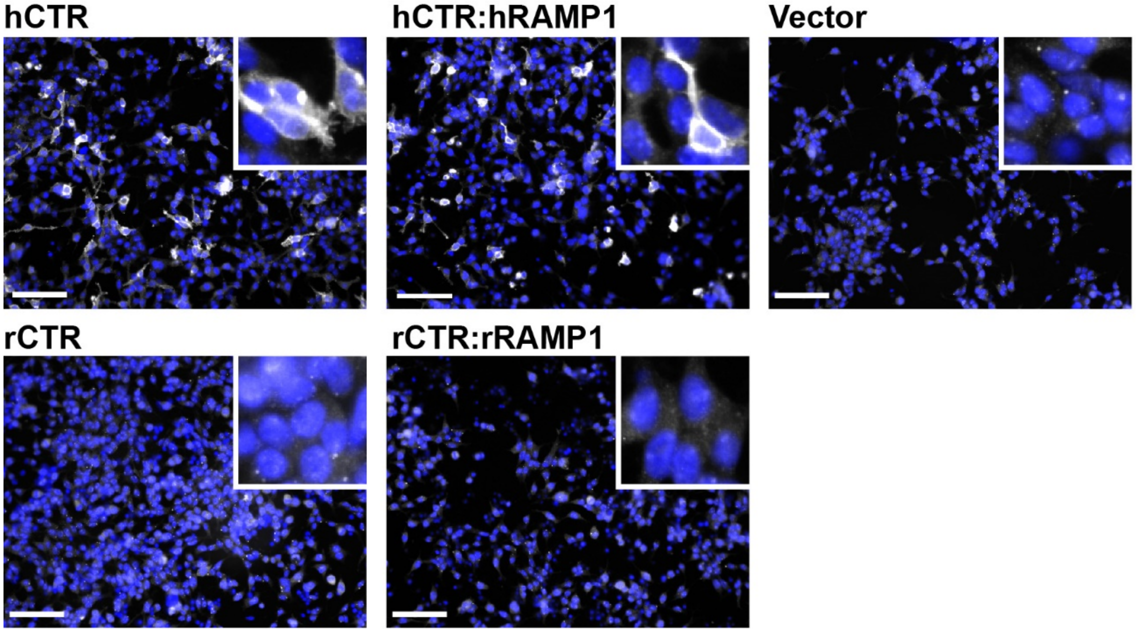

Supplementary Figure 9. Immunocytochemical staining of transfected HEK293S cells using anti-CTR pAb230500 antibody (5 µg/ml). Immunoreactive staining is shown in greyscale, and nuclear DAPI staining is shown in blue. h = human; r = rat. Scale bar = 100 µm. Images are representative of three independent experiments in duplicate wells. The brightness and contrast of these images has been enhanced uniformly for presentation purposes.

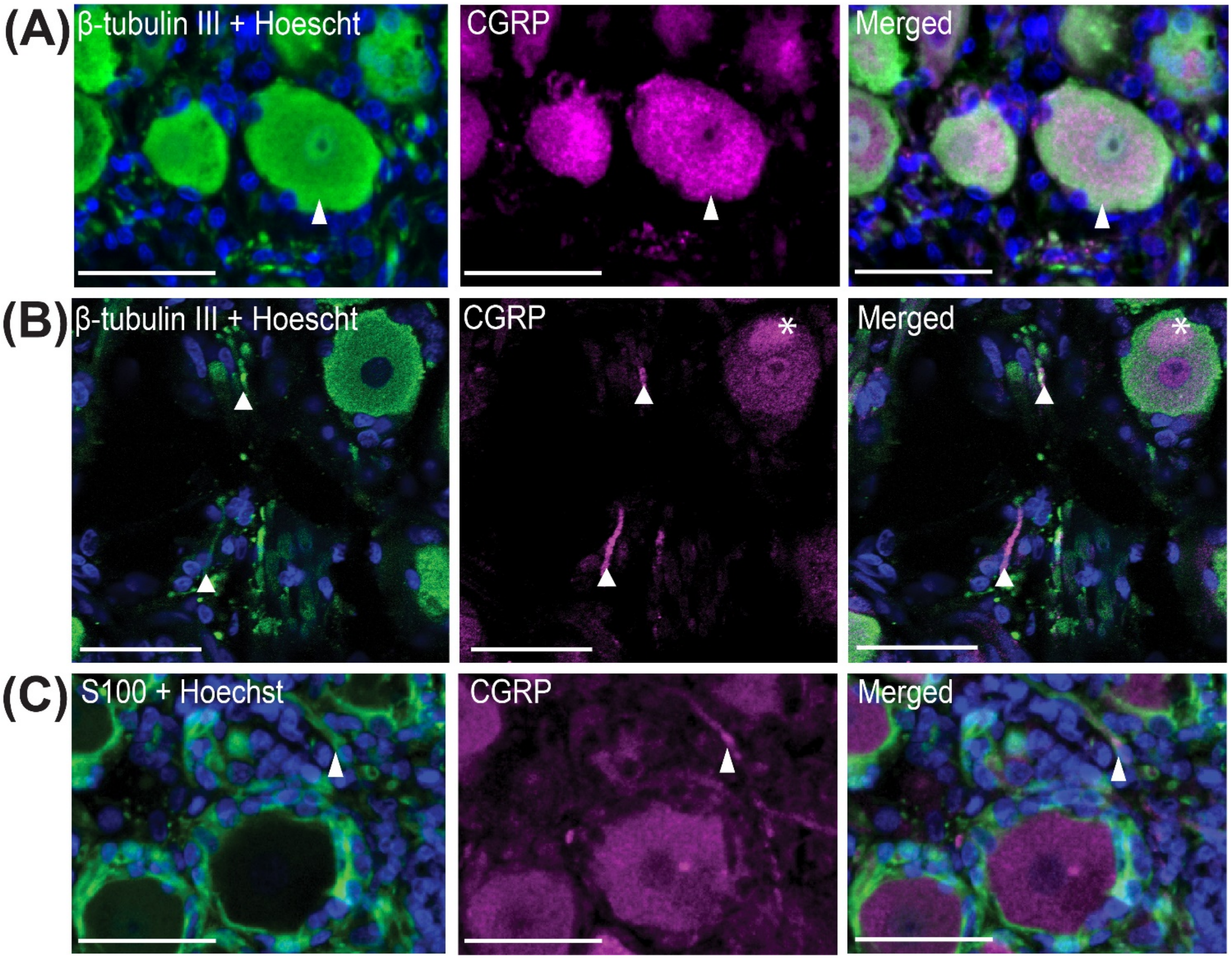

Supplementary Figure 10. Immunohistochemical localization of CGRP (pAb36001, 10 µg/ml) with β-tubulin III (1.2 µg/ml) and S100 (5 µg/ml) in human TG (Case 3A). Distribution of CGRP-LI in (A) puncta or (B, C) fibers relative to β tubulin III or S100. Filled arrowheads indicate examples of positive staining. *indicates examples of autofluorescence due to lipofuscin. Scale bar = 50 µm. Image brightness and contrast were adjusted for presentation purposes and merged in FIJI. Images are representative of four human cases.

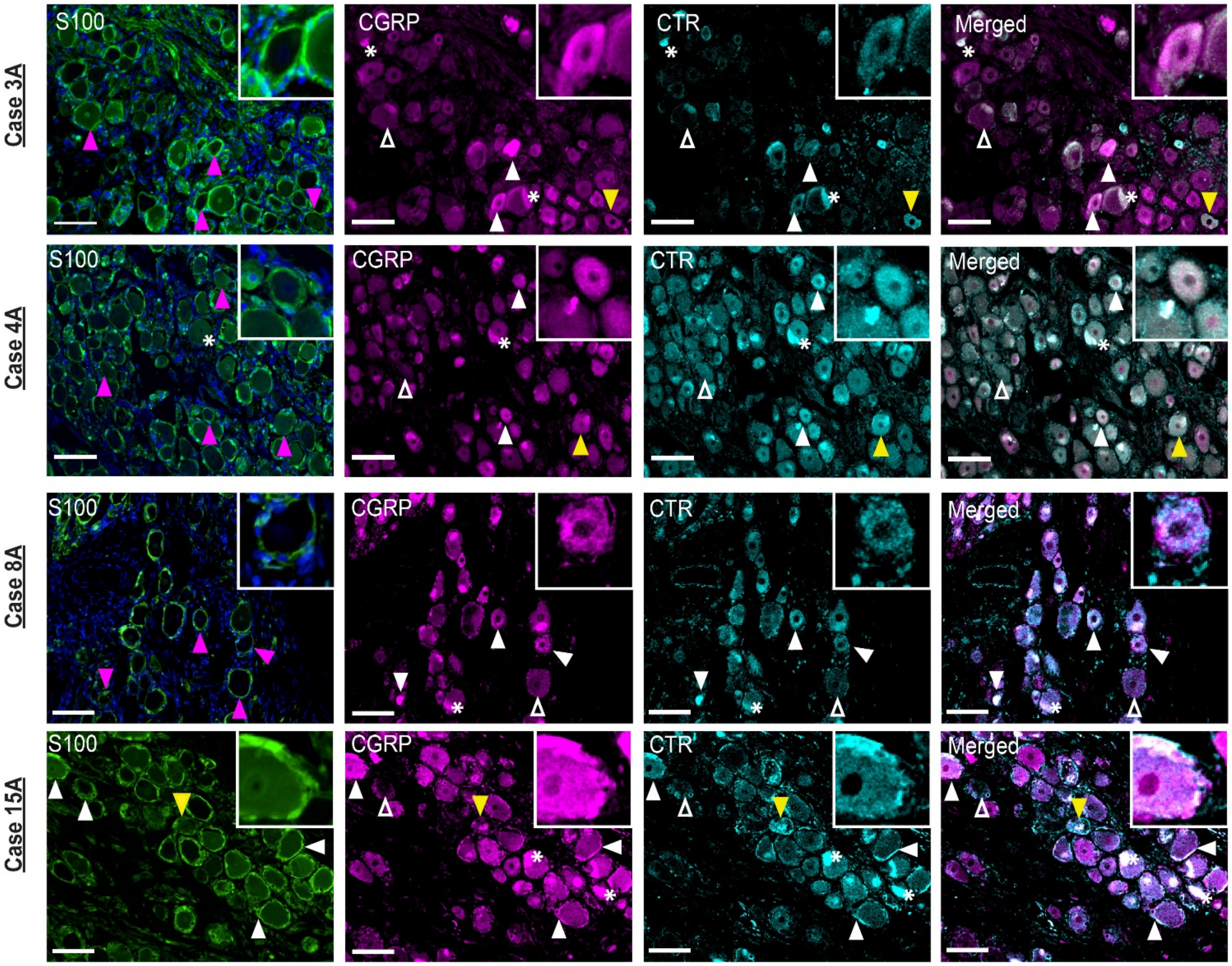

Supplementary Figure 11. Immunohistochemical localization of CGRP (pAb36001, 10 µg/ml) and CTR (mAb31-01, 4 µg/ml) with S100 (5 µg/ml) in human TG. Filled arrowheads indicate examples of positive staining; empty arrowheads indicate examples of an absence of staining. Magenta arrows indicate expression of S100 in glia surrounding neurons expressing, but not colocalizing with, CGRP or CTR; white arrowheads indicate colocalization; yellow arrowheads indicate expression in adjacent neurons. *indicates examples of autofluorescence due to lipofuscin. Scale bar = 100 µm. Image brightness and contrast were adjusted for presentation purposes and merged in FIJI. Images are representative of each of the four individual human cases.

2.2 Tables

Supplementary Table 1: Details of the antibodies used in this study

Catalogue number and supplier	RRID	Antigen	Species raised in	Poly or monoclonal	Dilution, µg/ml	Secondary
**Transfected HEK293S cells – CTR antibody validation**						
31-01, Welcome Receptor Antibodies Pty	AB_530746	hCTR C-terminal DIPIYICHQEPRNEPANN	Mouse	Monoclonal	1:500, 4 µg/ml	1:200 Donkey anti-mouse AF647 (A31571, ThermoFisher)
PA1-84457, ThermoFisher	AB_2228378	rCTR C-terminal proprietary	Rabbit	Polyclonal	1:100, 10 µg/ml	1:200 Donkey anti-rabbit AF647 (A31573, ThermoFisher)
Ab230500, Abcam	None	hCTR C-terminal proprietary between residue 400-478	Rabbit	Polyclonal	1:100, 5 µg/ml	1:200 Donkey anti-rabbit AF647 (A31573, ThermoFisher)
BIO 901503, Biolegend	AB_256005	HA, CYPYDVPDYASL	Mouse	Monoclonal	1:1000, 1 µg/ml	1:200 Donkey anti-mouse AF488 (A32766, ThermoFisher)
F1804, Sigma	AB_262044	Myc, EQKLISEEDL	Mouse	Monoclonal	1:250, 0.8 µg/ml	1:200 Donkey anti-mouse AF488 (A32766, ThermoFisher)
**Immunoblotting (Dot blots) – CGRP antibody validation**						
Ab36001, Abcam	AB_725807	Rat αCGRP 22-37	Goat	Polyclonal	1:1000, 5 µg/ml	1:1000 Donkey anti-goat HRP (A16005, ThermoFisher)
C8198, Sigma	AB_259091	Full length rat αCGRP	Rabbit	Whole antiserum	1:10,000, unknown	1:1000 Goat anti-rabbit HRP (A16110, ThermoFisher)
Ab81887, Abcam	AB_1658411	Full length rat αCGRP	Mouse	Monoclonal	1:500, 2 µg/ml	1:1000 Goat anti-mouse HRP (A16072, ThermoFisher)
ABS 026-05-02, Invitrogen	AB_1073000	Human αCGRP 1-18	Mouse	Monoclonal	1:500, 2.12 µg/ml	1:1000 Goat anti-mouse HRP (A16072, ThermoFisher)
**Rat pancreas and TG histology – CGRP antibody validation**						
Ab36001, Abcam	AB_725807	Rat αCGRP 22-37	Goat	Polyclonal	1:500, 10 µg/ml	1:200 Donkey anti-goat AF647 (A32849, ThermoFisher)
C8198, Sigma	AB_259091	Full length rat αCGRP	Rabbit	Whole antiserum	1:4000, unknown	1:200 Donkey anti-rabbit AF647 (A31573, ThermoFisher)

Ab81887, Abcam	AB_1658411	Full length rat αCGRP	Mouse	Monoclonal	1:100, 10 µg/ml	1:200 Donkey anti-mouse AF647 (A31571, ThermoFisher)
ABS 026-05-02, Invitrogen	AB_1073000	Human αCGRP 1-18	Mouse	Monoclonal	1:400, 2.65 µg/ml	1:200 Donkey anti-mouse AF647 (A31571, ThermoFisher)
**Mouse TG histology – Colocalization of CTR and CGRP**						
188, Welcome Receptor Antibodies Pty	AB_297696	rCTR C-terminal GLPIYICHQEPRNPPVSNN	Rabbit	Polyclonal	1:100, 20 µg/ml	1:200 Donkey anti-rabbit AF647 (A31573, ThermoFisher)
Ab36001, Abcam	AB_725807	Rat αCGRP 22-37	Goat	Polyclonal	1:500, 10 µg/ml	1:200 Donkey anti-goat AF555 (A21432, ThermoFisher)
T8578, Sigma	AB_1841228	β tubulin III	Mouse	Monoclonal	1:500, 2 µg/ml	1:200 Donkey anti-mouse AF488 (A32766, ThermoFisher)
N0142, Sigma	AB_477257	NF200	Mouse	Monoclonal	1:200, 72.5 µg/ml	1:200 Donkey anti-mouse AF488 (A32766, ThermoFisher)
**Rat TG histology – Colocalization of CTR and CGRP**						
188, Welcome Receptor Antibodies Pty	AB_297696	rCTR C-terminal GLPIYICHQEPRNPPVSNN	Rabbit	Polyclonal	1:200, 10 µg/ml	1:200 Donkey anti-rabbit AF647 (A31573, ThermoFisher)
Ab36001, Abcam	AB_725807	Rat αCGRP 22-37	Goat	Polyclonal	1:500, 10 µg/ml	1:200 Donkey anti-goat AF555 (A21432, ThermoFisher)
T8578, Sigma	AB_1841228	β tubulin III	Mouse	Monoclonal	1:500, 2 µg/ml	1:200 Donkey anti-mouse AF488 (A32766, ThermoFisher)
N0142, Sigma	AB_477257	NF200	Mouse	Monoclonal	1:200, 72.5 µg/ml	1:200 Donkey anti-mouse AF488 (A32766, ThermoFisher)
**Human TG histology – Colocalization of CTR and CGRP**						
31-01, Welcome Receptor Antibodies Pty	AB_530746	hCTR C-terminal DIPIYICHQEPRNEPANN	Mouse	Monoclonal	1:250, 4 µg/ml	1:250 Donkey anti-mouse AF647 (A31571, ThermoFisher)
Ab36001, Abcam	AB_725807	Rat αCGRP 22-37	Goat	Polyclonal	1:500, 10 µg/ml	1:250 Donkey anti-goat AF555 (A21432, ThermoFisher)
T2200, Sigma	AB_262133	β tubulin III	Rabbit	Polyclonal	1:500, 1.2 µg/ml	1:250 Donkey anti-rabbit AF488 (A21206, ThermoFisher)
Ab52642, Abcam	AB_882426	Anti-S100β	Rabbit	Monoclonal	1:200, 4.9 µg/ml	1:250 Donkey anti-rabbit AF488 (A21206, ThermoFisher)
**Western blotting – TG lysate**						

188, Welcome Receptor Antibodies Pty	AB_297696	rCTR C-terminal GLPIYICHQEPRNPPVSNN	Rabbit	Polyclonal	1:500, 4 µg/ml	1:2000 Goat anti-rabbit HRP (A16110, ThermoFisher)
8B9, Welcome Receptor Antibodies Pty	AB_2891126	rCTR C-terminal GLPIYICHQEPRNPPVSNN	Mouse	Monoclonal	1:500, 2 µg/ml	1:2000 Goat anti-mouse HRP (A16072, ThermoFisher)
31-01, Welcome Receptor Antibodies Pty	AB_530746	hCTR C-terminal DIPIYICHQEPRNEPANN	Mouse	Monoclonal	1:500, 2 µg/ml	1:2000 Goat anti-mouse HRP (A16072, ThermoFisher)

Supplementary Table 2. Details of rats and mice used for immunohistochemistry and western blotting.

**Rats**				**Mice**			
**Animal**	**Sex**	**Weight (g)**	**Age (weeks)**	**Animal**	**Sex**	**Weight (g)**	**Age (weeks)**
**Immunohistochemistry/Image analysis**							
Rat 1	Female	272	19	Mouse 1	Female	23.8	16
Rat 2	Male	490	19	Mouse 2	Female	27.8	16
Rat 3	Male	517	19	Mouse 3	Male	24.3	10
Rat 4	Female	258	14	Mouse 4	Male	23.8	10
Rat 5	Female	274	14	Mouse 5	Female	21.7	12
Rat 6	Male	657	23	Mouse 6	Male	28.9	12
**Western blot**							
Rat 7	Female	268	13	Mouse 7	Female	24.6	22
Rat 8	Female	256	13	Mouse 8	Male	26.8	13
Rat 9	Male	415	11	Mouse 9	Male	26.7	14

Supplementary Table 3. Details of human cases used for immunohistochemistry and western blotting.

Case	Sex	Age (years)	Tissue	Cause of death	Clinical notes
**Immunohistochemistry**					
15A	Male	94	TG	Bronchopneumonia	Slow, steady dementia
3A	Female	92	TG	Aspiration pneumonia/respiratory failure	Mild, slowly progressive dementia
4A	Male	87	TG	Myocardial infarction	No cognitive decline reported
8A	Male	87	TG	Congestive cardiac failure	Mild confusion/Dementia, No Parkinson symptoms
**Western blotting**					
1543	Male	17	TG	Motor vehicle accident	None
1322	Male	16	TN	Motor vehicle accident	None

Supplementary Table 4: Western blot protein loading.

**mAb31-01 Antibody characterization in transfected HEK-293S cells**	
Human CTR HEK293S lysate	10 µg
pcDNA HEK293S lysate	10 µg
**mAb8B9 mouse and rat tissue western blot**	
Mouse and rat CTR HEK293S lysate	10 µg
Mouse or rat TG	20 µg
pcDNA HEK293S lysate	10 µg
**pAb188 mouse and rat tissue western blot**	
Mouse CTR HEK293S lysate	0.3 µg
Rat CTR HEK293S lysate	0.1 µg
Mouse or rat TG	20 µg
pcDNA HEK293S lysate	0.1 or 0.3 µg
**pAb188 mouse and rat tissue western blot – higher protein loading (Figure S6)**	
Mouse or rat CTR HEK293S lysate	10 µg
Mouse or rat TG	20 µg
pcDNA HEK293S lysate	10 µg
**mAb31-01 human TG and TN tissue western blot**	
Human CTR HEK293S lysate	10 µg
Human TG or TN lysate	20 µg
pcDNA HEK293S lysate	10 µg

Supplementary Table 5: Results summary for validation of the CGRP antibodies.

Antibody	Immunoblots*					Pancreas histology	TG histology
	**hAmy**	**rAmy**	**hαCGRP**	**rαCGRP**	**Selectivity****	**Islets stained**	**Neurons stained**
Ab36001	N<br>(100 µg)	N<br>(100 µg)	10 ng	10 ng	>10,000	N***	Y
C8198	N<br>(100 µg)	N<br>(100 µg)	50 ng	10 ng	>10,000	N***	Y
Ab81887	N<br>(100 µg)	N<br>(100 µg)	25 ng	10 ng	>10,000	N***	Y
ABS 026-05-02	N<br>(100 µg)	100 µg	25 ng	10 ng	~10,000	Y***	Y

N, no signal in immunoblot or staining in histology. n.d. not determined. Y, yes staining was observed. *For immunoblots, the amounts of peptide refer to the lowest quantity that was reliably detected, with the value in parentheses being the highest quantity tested for that antibody/peptide combination in cases where there was no reliable detection. **Conservative estimate of selectivity is based on the ratio between the lowest amount of rat amylin and rat CGRP detected in immunoblots. ***Pearl-like staining resembling neuronal fibers observed.

References:

Neris, R.L.S., Kaur, A., and Gomes, A.V. (2020). Incorrect Molecular Weights due to inaccurate Prestained Protein Molecular Weight Markers that are used for Gel Electrophoresis and Western Blotting. *BioRxiv*. doi: https://doi.org/10.1101/2020.04.03.023465.
